# Exposure to heavy metals in *utero* and autism spectrum disorder at age 3: a meta-analysis of two longitudinal cohorts of siblings of children with autism

**DOI:** 10.1186/s12940-024-01101-2

**Published:** 2024-07-05

**Authors:** John F. Dou, Rebecca J. Schmidt, Heather E. Volk, Manon M. Nitta, Jason I. Feinberg, Craig J. Newschaffer, Lisa A. Croen, Irva Hertz-Picciotto, M. Daniele Fallin, Kelly M. Bakulski

**Affiliations:** 1 University of Michigan, Ann Arbor, MI USA; 2 University of California Davis, Davis, CA USA; 3 Johns Hopkins University, Baltimore, MD USA; 4 Penn State University, State College, PA USA; 5 Division of Research, Kaiser Permanente Northern California, Oakland, CA USA; 6 Rollins School of Public Health, Emory University, Atlanta, GA USA

**Keywords:** Metals exposure, Autism spectrum disorder, Pregnancy cohort, Epidemiology, Cadmium, ExWAS

## Abstract

**Background:**

Autism spectrum disorder (ASD) is a prevalent and heterogeneous neurodevelopmental disorder. Risk is attributed to genetic and prenatal environmental factors, though the environmental agents are incompletely characterized.

**Methods:**

In Early Autism Risk Longitudinal Investigation (EARLI) and Markers of Autism Risk in Babies Learning Early Signs (MARBLES), two pregnancy cohorts of siblings of children with ASD, urinary metals concentrations during two pregnancy time periods (< 28 weeks and ≥ 28 weeks of gestation) were measured using inductively coupled plasma mass spectrometry. At age three, clinicians assessed ASD with DSM-5 criteria. In an exposure-wide association framework, using multivariable log binomial regression, we examined each metal for association with ASD status, adjusting for gestational age at urine sampling, child sex, age at pregnancy, race/ethnicity and education. We meta-analyzed across the two cohorts.

**Results:**

In EARLI (*n* = 170) 17% of children were diagnosed with ASD, and 44% were classified as having non-neurotypical development (Non-TD). In MARBLES (*n* = 231), 21% were diagnosed with ASD, and 14% classified as Non-TD. During the first and second trimester period (< 28 weeks), having cadmium concentration over the level of detection was associated with 1.69 (1.08, 2.64) times higher risk of ASD, and 1.29 (0.95, 1.75)times higher risk of Non-TD.

A doubling of first and second trimester cesium concentration was marginally associated with 1.89 (0.94, 3.80) times higher risk of ASD, and a doubling of third trimester cesium with 1.69 (0.97, 2.95) times higher risk of ASD.

**Conclusion:**

Exposure in utero to elevated levels of cadmium and cesium, as measured in urine collected during pregnancy, was associated with increased risk of developing ASD.

**Supplementary Information:**

The online version contains supplementary material available at 10.1186/s12940-024-01101-2.

## Background

Autism Spectrum Disorder (ASD) presents a major public health concern. ASD is a neurodevelopmental disorder characterized by impairments in social communication, social interaction, and restrictive and repetitive behavioral patterns and interests [1]. In the United States, 1 in 36 children are affected by ASD, with the prevalence among males 3.8 times greater than among females [2]. Individuals with ASD and their families face significant social and financial burdens, with higher costs for individuals with more severe ASD [3]. The social cost of ASD was greater than $7 trillion between the years 1990 – 2019 and is projected to be an additional $4 to $15 trillion by 2029 [4]. Increases in the prevalence of diagnosed ASD have been attributed to increasing social awareness [5], changes to diagnostic criteria [6], and participation in early intervention services [7]. However, the full source of this increase is largely unknown, suggesting incidence may be rising. Environmental exposures may play a role in this increase. Understanding modifiable risk factors for ASD could play a major role in guiding public health interventions.

Metals exposures are potential modifiable risk factors in ASD. Among children diagnosed with ASD relative to controls, higher childhood blood levels of arsenic [8], mercury [8–10], lead [10–12], and cadmium [13] have been observed. Although these findings are suggestive, exposure to metals was measured after ASD diagnosis, and it is not known if elevated exposure levels preceded ASD. Studies examining metals exposure during pregnancy and ASD diagnosis are less common. In the United States, persons of childbearing age experience widespread environmental exposure to metals, and higher concentrations have been observed in pregnant compared to non-pregnant people [14, 15]. Important neurodevelopmental processes occur during the in utero period [16], and exposure to environmental factors such as metals are suggested to have a role in ASD etiology [17–19]. Poorer performance on social and behavioral tests among children at age 3 was associated with elevated manganese levels in infant toenails and arsenic in toenails of pregnant persons [20], and blood lead levels of pregnant persons [21]. In contrast, elevated copper levels in urine or blood collected during pregnancy was associated with decreased behavior problems assessed in children aged 3–7 years [22]. Additionally, elemental biomarkers measured in hair at one month of age, including essential and non-essential metals, have been shown to be predictive of future ASD diagnosis [23]. One nested case–control study in the Norwegian Mother, Father, and Child Cohort Study linked with the Norwegian Patient Registry examined blood metals concentrations during pregnancy, finding elevated arsenic, cadmium, and manganese were associated with ASD, and lower levels of cesium, copper, mercury, and zinc were associated with ASD [24]. There are limited studies on prenatal metals exposure and ASD, and more prospective cohorts with exposure measures of multiple metals are needed.

Given the previous heterogeneous inclusion of and approaches for different metals with ASD, we were motivated to perform a consistent discovery analysis across multiple metals. This study was conducted in two pregnancy cohorts of siblings of children with ASD, the Early Autism Risk Longitudinal Investigation (EARLI) and the Markers of Autism Risk in Babies—Learning Early Signs (MARBLES) study. The goal of this study was to use an environment-wide association study (ExWAS) framework to screen a panel of twenty-two metals measured during two time periods of pregnancy for associations with ASD diagnosis in children at age 3 years. The ExWAS is a data-driven exploratory approach to identify a subset of exposure measures most strongly associated with a trait [25] for follow-up in toxicologic or epidemiologic designs [26]. The design is inspired by the genome-wide association framework, and involves testing pairwise relationships between exposures and the outcome, producing interpretable results while accounting for multiple comparisons [25].

## Methods

### Study sample

The Early Autism Risk Longitudinal Investigation (EARLI) and Markers of Autism Risk Learning Early Signs (MARBLES) studies are prospective pregnancy cohorts to study autism etiology [27, 28]. These studies recruited parents of children with clinically confirmed ASD (probands) who were early in a subsequent pregnancy or were trying to become pregnant. Siblings of children with ASD are more likely to have a diagnosis of ASD or other developmental delays [29, 30]. In EARLI there was 232 participants with a subsequent child (sibling) born during the study between November 2009 and March 2012. In MARBLES there was 389 enrolled participants that gave birth to 425 subsequent children (sibling) between December 1, 2006 and July 1, 2016.

### Covariate and outcome assessment

Demographics, pregnancy behaviors, and medical history were all collected via questionnaire at enrollment. Clinicians assessed children born during the study (siblings) at age three years using DSM-5 criteria. Children were categorized into three groups: typically developing, ASD, or non-typical development. Outcome categorization, based on a previously published algorithm using the Autism Diagnostic Observation Schedule (ADOS) and the Mullen Scales of Early Learning (MSEL) [31], has been described in these cohorts previously [32, 33]. In brief, those who met diagnostic DSM-5 criteria and ADOS scores over the cutoff were categorized in the ASD group. Those that did not meet diagnostic criteria, but had ADOS scores within three points of the cutoff or MSEL scores 1.5 to 2 standard deviations below average, were categorized in the non-typical development group. Finally, the typical development group did not meet diagnostic criteria for ASD and were not categorized in the non-typical development group.

### Exposure assessment

Urinary metals measures generally reflect acute or recent exposure, with exceptions such as cadmium reflecting cumulative exposure, and some metals such as lead and manganese have unclear urinary measure relations [34]. Timeframes of exposure reflected in urinary metals measures are summarized in Table S1. Urine samples were collected at two time periods during pregnancy: trimester 1/ trimester 2 (T1/T2) collected between 5 to less than 28 weeks of pregnancy (approximate mean 19 weeks), and trimester 3 (T3) collected at 28 to 40 weeks of pregnancy (approximate mean 32 weeks). Urinary concentrations of a panel of metals were measured using inductively coupled plasma mass spectrometry by NSF International (Centers for Disease Control and Prevention method 3018.3, with modifications for the expanded metals panel and the Thermo Scientific iCAP RQ instrument). Metals measured include antimony, arsenic, barium, beryllium, cadmium, cesium, chromium, cobalt, copper, lead, manganese, mercury, molybdenum, nickel, platinum, selenium, thallium, tin, tungsten, uranium, vanadium, and zinc. Samples for both cohorts were randomized together into two laboratory runs and runs had variable limits of detection (LOD) (Table S2). To assess urinary dilution, specific gravity was measured by NSF International using an ATAGO handheld digital refractometer model PAL-10S.

Used in a sensitivity analysis, blood concentrations during pregnancy of cadmium, manganese, lead, selenium, and total mercury were also measured in EARLI. Venous blood samples were collected from pregnant participants in trace metal free EDTA tubes. Blood samples from the first study visit (*n* = 215) were used. Metal concentrations in blood samples were measured by inductively coupled dynamic reaction cell plasma mass spectrometry by the US Centers for Disease Control and Prevention (ELAN DRC II, PerkinElmer Norwalk, CT) (method DLS 3016.8, Centers for Disease Control and Prevention). Micro-clotting of the archived blood prevented measures in half of samples, leaving *n* = 92 with measured concentrations, as well as full data on outcome and covariates.

### Statistical analyses

We used R statistical software (version 4.0.2) for statistical analysis. Code to produce analyses is available (https://github.com/bakulskilab /Urine-Metals-ASD). The percentage of samples above LOD were computed with all samples (regardless of pregnancy time period or cohort) pooled together. Metals with less than approximately 10% of samples above the LOD were dropped from analysis (beryllium, platinum, tungsten, uranium, vanadium). Metals with less than 75% of samples above the LOD (antimony, cadmium, chromium, lead) were treated as binary variables, based on whether a sample was above or below the LOD. For the rest of the metals, concentrations were used as continuous variables. We substituted all urinary metals measures quantitated with values below the LOD with the value of the LOD/square root of two [35]. Metal concentrations were adjusted for specific gravity by multiplying concentrations by the ratio of [the median specific gravity – 1] and [sample specific gravity – 1] [36]. We then log_2_ transformed the adjusted concentrations. Outlier metals concentrations > 5 standard deviations from the mean were dropped from analyses. The number of samples dropped per metal are listed in Table S3.

We separated urinary measures into T1/T2 and T3 pregnancy time periods. Samples with gestational age at collection < 28 weeks were considered T1/T2, and samples with gestational age at collection ≥ 28 weeks were considered T3. For those with two measures categorized in the same time period, the sample with gestational age at collection furthest from 28 weeks was used (lowest gestational age for T1/T2, highest gestational age for T3). Distribution of gestational age at urine sample collection are shown in Figure S1. We compared exposure levels in T1/T2 and T3 pregnancy with Spearman correlation tests.

We applied several exclusion criteria, summarized in Figure S2. We excluded individuals involved in a multiple birth (*n* = 7 in EARLI, *n* = 5 in MARBLES), related siblings from non-multiple births (selecting one randomly to keep, *n* = 11 individuals), and those smoking during pregnancy (*n* = 7 in EARLI, *n* = 9 in MARBLES). We excluded samples missing gestational age at collection (3 samples, from individuals that still had a valid sample from different time period), and sample closest to 28 weeks of pregnancy if two were collected during same time period (only in EARLI, 12 samples in T1/T2, 10 samples in T3) EARLI had 137 mothers with metals measures from two time periods, and 33 mothers with a measure from one time period. MARBLES had 142 mothers with metals measures from two time period, and 89 mothers with a measure from one time period. In EARLI, 3 individuals were missing education information, resulting in *n* = 151 for the early pregnancy period, and *n* = 156 for the late pregnancy period. In MARBLES, 11 individuals were missing ASD status, resulting in *n* = 151 for the early pregnancy period, and *n* = 222 for the late pregnancy period.

We calculated univariate descriptive statistics on each cohort using mean and standard deviation for continuous variables and count and frequency for categorical variables. The distributions of metal concentrations were described using mean, median, standard deviation, interquartile range, and the number and percent above the limit of detection. We calculated Spearman correlation of metals concentrations within each cohort. Separately for each cohort, we compared the bivariate sample characteristics by neurodevelopmental outcome (ASD, non-typically developing, typically developing) using ANOVA tests for continuous variables and chi-square tests for categorical variables.

Models included individuals with non-missing data on urinary metal concentration, ASD status, and covariates of interest. To estimate the adjusted associations between urinary metals concentration in pregnancy and neurodevelopmental status, we used log binomial models to estimate risk ratios (RR). Due to convergence issues, we used the delta-method normal approximations for fitting models using the epitools package [37]. In the ExWAS framework, we estimated the association of each metal (in separate models) with ASD status relative to the typically developing group using the log_2_ transformed concentrations, adjusting for gestational age at urine sampling, child sex, and the following characteristics of pregnant participants: age, education, self-reported race/ethnicity. We also tested metals associations with non-typically developing status (typically developing as reference) in separate log binomial models.

Models were fit separately for each cohort, then meta analyzed together using the inverse variance method in the R meta package [38]. We reported risk ratios and 95% confidence intervals (95% CI) for each association and visualized the results using forest plots. For metals that were modeled continuously, since concentrations were log_2_-transformed, the reported associations are for a doubling in concentration. For metals that were modeled as binary, we reported the RR for above versus below the LOD. To account for multiple comparisons, we also reported false discovery rate adjusted p-values.

We performed several sensitivity analyses to assess the robustness of our findings. Since runs for metals measures had variable LODs, which impacts binary categorization and imputation for values below LOD, we ran models adjusted for batch. We also performed multivariable logistic regression for each of our models to generate adjusted odds ratios (OR) that may be compared to the risk ratios and to prior findings in the literature. Lastly, we performed analyses on the subset of EARLI samples with blood metals measures during pregnancy available and compared the findings to the findings in urinary metals.

## Results

### Sample descriptive statistics

**Fig.1:**
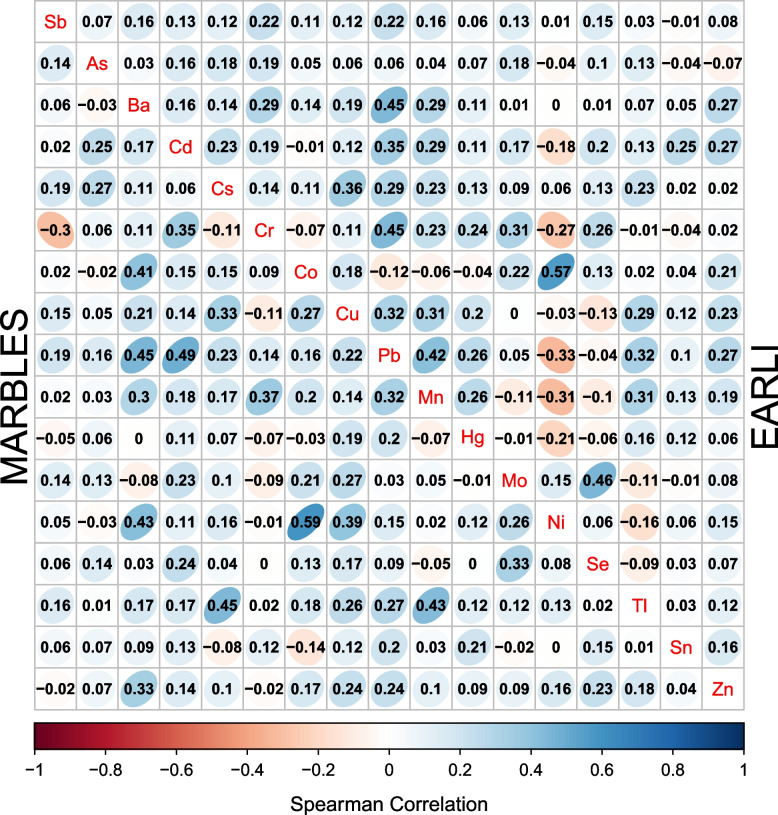
Spearman correlations of urinary metals concentrations, measured during trimester 1 or 2 pregnancy, stratified by cohort. The upper right triangle shows the EARLI cohort. The lower left triangle shows the MARBLES cohort. Metals are represented by their chemical symbol along the diagonal

At the T1/T2 time period, urinary metal concentrations were above the LOD in greater than 75% of the samples for 13 metals in each cohort (arsenic, barium, cesium, cobalt, copper, manganese, mercury, molybdenum, nickel, selenium, thallium, tin, and zinc) (Table S4). In both EARLI and MARBLES, cobalt (Co) and nickel (Ni) concentrations had the strongest correlation (Spearman *r* = 0.57 in EARLI, *r* = 0.59 in MARBLES) (Fig. 1). At the T3 pregnancy time period, urinary metal concentrations were above the limit of detection in slightly less than 75% of the sample for manganese, mercury, and tin (Table S5), however they were modelled as continuous as decisions were based on LOD percentages from all samples pooled. In T3 pregnancy, cobalt and nickel remained the strongest correlated metals in MARBLES (Spearman *r* = 0.74), but not in EARLI. In both cohorts, lead and copper (*r* = 0.43 in EARLI, *r* = 0.49in MARBLES) as well as lead and manganese (*r* = 0.45 in both) were correlated (Figure S3).

In EARLI, education and child sex assigned at birth were associated with child neurodevelopmental status. The typically developing group were birthed from individuals with higher levels of education (72% with college degree), compared to the non-typically developing (54%) and ASD groups (45%). The typically developing and non-typically developing groups had a similar proportion of males (43% and 49%) but lower proportion than the ASD group (83%). In MARBLES, compared to the typically developing group (52% male) and the non-typically developing group (55% male), ASD (67% male) had higher proportion of males (Table 1). In the T1/T2 pregnancy time period there were metals concentrations available from 151 urine samples in EARLI (63 typically developing, 67 non-typically developing, 21 ASD) and 151 in MARBLES (100 typically developing, 17 non-typically developing, 34 ASD) (Table S6). At the T3 pregnancy time period, there were 156 samples with urinary metal concentrations available in EARLI (63 typically developing, 68 non-typically developing, 25 ASD) and 222 in MARBLES (141 typically developing, 33 non-typically developing, 48 ASD) (Table S7).

**Table 1:** Pregnant person and child characteristics of participants in the analytic sample with urinary metals measures in either trimester 1&2 (T1/T2) (< 28 weeks gestation) or trimester 3 (T3). Distributions of categorical variables are compared with a chi-square test and continuous variables are compared with ANOVA test

EARLI cohort	Typically developing	Non-typically developing	Autism spectrum disorder	*P*-value
	*N* = 67	*N* = 74	*N* = 29	
Education				0.022
College Degree	48 (72%)	40 (54%)	13 (45%)	
No Degree	19 (28%)	34 (46%)	16 (55%)	
Age	34.99 (4.8)	32.97 (4.7)	33.72 (3.8)	0.092
Self Report Race/Ethnicity				0.3
Asian or Pacific Islander	9 (13%)	12 (16%)	4 (14%)	
Black	4 (6.0%)	11 (15%)	3 (10%)	
Hispanic	7 (10%)	12 (16%)	6 (21%)	
Other/Multiracial	2 (3.0%)	6 (8.1%)	2 (6.9%)	
White	45 (67%)	33 (45%)	14 (48%)	
Infant Sex				0.001
Female	38 (57%)	38 (51%)	5 (17%)	
Male	29 (43%)	36 (49%)	24 (83%)	
Weeks Gestation at Collection T1/T2	18.69 (4.8)	17.83 (5.8)	16.98 (7.1)	> 0.9
No sample in period	4	7	8	
Weeks Gestation at Collection T3	33.07 (2.9)	33.13 (2.7)	33.44 (3.3)	> 0.9
No sample in period	4	6	4	
MARBLES cohort	Typically developing	Non-typically developing	Autism spectrum disorder	*P*-value
	*N* = 149	*N* = 33	*N* = 49	
Education				0.3
College Degree	84 (56%)	15 (45%)	22 (45%)	
No Degree	65 (44%)	18 (55%)	27 (55%)	
Age	34.06 (4.7)	34.01 (4.4)	34.84 (5.1)	0.5
Self Report Race/Ethnicity				0.3
Asian or Pacific Islander	24 (16%)	6 (18%)	6 (12%)	
Black	3 (2.0%)	3 (9.1%)	4 (8.2%)	
Hispanic	34 (23%)	9 (27%)	12 (24%)	
Other/Multiracial	3 (2.0%)	0 (0%)	2 (4.1%)	
White	85 (57%)	15 (45%)	25 (51%)	
Infant Sex				0.2
Female	71 (48%)	15 (45%)	16 (33%)	
Male	78 (52%)	18 (55%)	33 (67%)	
Weeks Gestation at Collection T1/T2	19.36 (4.0)	18.86 (4.1)	19.06 (4.0)	0.8
No sample in period	49	16	15	
Weeks Gestation at Collection T3	31.41 (3.1)	31.39 (3.0)	31.36 (3.3)	> 0.9
No sample in period	8	0	1	

*Acronyms: Early Autism Risk Longitudinal Investigation (EARLI), Markers of Autism Risk in Babies-Learning Early Signs (MARBLES), trimester (T)*

For participants with two time periods, correlation between the two were strongest for measured cesium (*r* = 0.53 in EARLI, *r* = 0.58 in MARBLES), mercury (*r* = 0.50 in EARLI, *r* = 0.43 in MARBLES), tin (*r* = 0.59 in EARLI, *r* = 0.53in MARBLES), and zinc (*r* = 0.56 in EARLI, *r* = 0.51 in MARBLES). Cross time period correlation was weakest for cobalt (*r* = 019 in EARLI, *r* = 0.30 in MARBLES), manganese (*r* = 0.12 in EARLI, *r* = -0.07 in MARBLES), and molybdenum (*r* = 0.3 in EARLI, *r* = 0.09 in MARBLES) (Table S8).

### Urinary metal association with autism spectrum disorder status

**Fig. 2:**
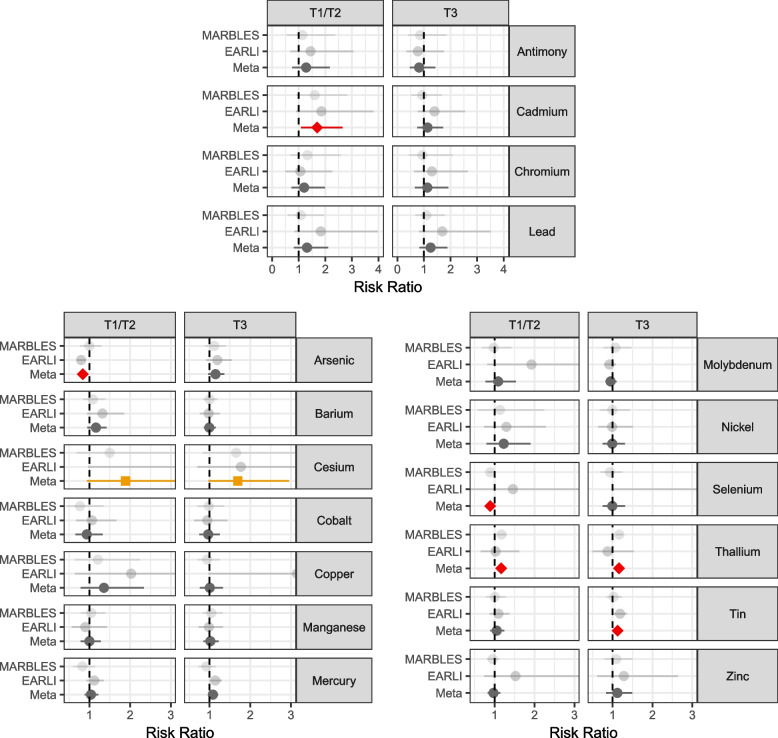
Adjusted risk ratios for the associations between urinary metals concentrations measuring during pregnancy and risk of autism spectrum disorder, relative to typically developing. Antimony, cadmium, chromium, and lead compare over limit of detection vs under the limit of detection for that metal. Remaining metals show the risk ratio for a doubling in metal concentration. Analyses were performed stratified by cohort (EARLI and MARBLES) and then meta-analyzed across cohorts. Red/diamond denotes a nominal meta-analysis *p*-value < 0.05, and orange/square a nominal meta-analysis *p*-value < 0.10

We examined associations between urinary metals in the T1/T2 pregnancy time period and ASD. In meta-analysis, comparing ASD to typical development, having urine cadmium concentration above the limit of detection was associated with 1.69 (95% CI 1.08, 2.64) times higher risk for ASD (EARLI RR = 1.85, 95% CI 0.90, 3.81; MARBLES RR = 1.60, 95% CI 0.91, 2.82). (Fig. 2**, **Table S9). A doubling in arsenic was associated with lower ASD risk (RR = 0.84, 95% CI 0.74, 0.94), driven by the EARLI cohort (EARLI RR = 0.80, 95% CI 0.69, 0.91; MARBLES RR = 1.0, 95% CI 0.77, 1.29). Similarly, selenium was associated with lower ASD risk (RR = 0.89, 95% CI 0.83,0.95), driven by the MARBLES cohort (EARLI RR = 1.46, 95% CI 0.37, 5.75; MARBLES RR = 0.88, 95% CI 0.83, 0.94). Thallium concentration doubling was associated with RR = 1.16 (95% CI 1.05, 1.28), with stronger effect in MARBLES (RR = 1.17, 95% CI 1.06, 1.30) than in EARLI (RR = 1.02, 95% CI 0.65, 1.61). Marginal associations were observed with cesium, where a doubling in urinary concentration was estimated to have RR = 1.89 (95% CI 0.94, 3.8). The associations for arsenic (FDR = 0.05), selenium (FDR = 0.01), and thallium (FDR = 0.05) reached FDR < 0.1 when adjusting for multiple comparisons. No associations were observed between the remaining urinary metal concentrations and ASD status at the T1/T2 pregnancy time period.

At the T3 pregnancy time period, we estimated the association between each metal concentration and ASD. Comparing ASD to typically developing in meta-analyses, a doubling in cesium was marginally associated with ASD (RR = 1.69, 95% CI 0.97, 2.95) in meta-analysis (EARLI RR = 1.77, 95% CI 0.70, 4.46.; MARBLES RR = 1.65, 95% CI 0.83, 3.31) (Fig. 2**, **Table S9). A doubling in thallium was associated with ASD with RR = 1.16 (95% CI 1.08,1.25), though effects were different between cohorts (EARLI RR = 0.87, 95% CI 0.50, 1.53.; MARBLES RR = 1.17, 95% CI 1.08, 1.26). A doubling in tin was associated with 1.13 (95% CI 1.01,1.26) times risk of ASD. The association with thallium reached FDR < 0.1. No associations were observed between the remaining urinary metal concentrations and ASD status at the T3 pregnancy time period.

### Urinary metal association with non-typically developing status

**Fig. 3:**
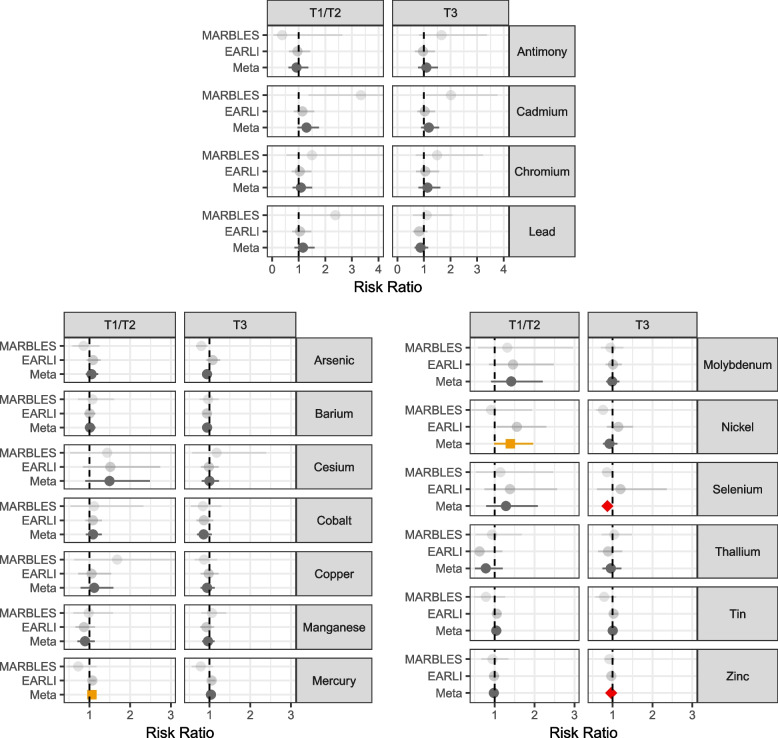
Adjusted risk ratios for the associations between urinary metals concentrations measuring during pregnancy and risk of non-typically developing, relative to typically developing. Antimony, cadmium, chromium, and lead compare over limit of detection vs under the limit of detection for that metal. Remaining metals show risk ratio for a doubling in metal concentration. Analyses were performed stratified by cohort (EARLI and MARBLES) and then meta-analyzed across cohorts. Red/diamond denotes a nominal meta-analysis *p*-value < 0.05, and orange/square a nominal meta-analysis *p*-value < 0.10

We repeated the adjusted regression analyses to estimate the association of T1/T2 pregnancy urinary metals and non-typically developing status. A marginal relationship with mercury and non-typical development was observed, where a doubling in concentration had estimated RR = 1.06 (95% CI 0.99, 1.14), driven by the EARLI cohort (EARLI RR = 1.07, 95% CI 1.00, 1.15; MARBLES RR = 0.73, 95% CI 0.46, 1.18). A doubling of nickel was marginally associated with 1.391.40 (95% CI 0.99, 1.96) times risk of non-typical development, also driven by the EARLI cohort (EARLI RR = 1.56, 95% CI 1.06, 2.30; MARBLES RR = 0.91, 95% CI 0.43, 1.93) (Fig. 3,Table S10). Though not statistically significant, having urine cadmium concentrations above the LOD was suggestive of elevated risk of non-typical development, with RR = 1.29 (95% CI 0.95, 1.75), and a doubling of cesium was suggestive of elevated non-typical development risk (RR = 1.49, 95% CI 0.90, 2.49). No associations were observed between the remaining urinary metal concentrations and non-typically developing status at the T1/T2 pregnancy time period.

We examined associations between non-typically developing and the T3 pregnancy metals measures. (Fig. 3, Table S10). A doubling of the essential metal selenium concentration was associated in meta-analysis with 0.87 (95% CI 0.76, 1.00) times lower risk of non-typically developing status, driven by precision of results in MARBLES and had opposite directions of effect by cohort (EARLI RR = 1.20, 95% CI 0.61, 2.36; MARBLES RR = 0.86, 95% CI 0.75, 0.99). A doubling of the essential metal zinc concentration was associated with 0.97 (95% CI 0.94, 0.99) times lower risk of non-typically developing status. No associations were observed between the remaining urinary metal concentrations and non-typically developing status at the T3 pregnancy time period.

### Pregnancy blood metal association with neurodevelopmental status

In EARLI, 92 blood samples collected during pregnancy had available covariate and blood metals measures (41 typically developing, 32 non- typically developing, 19 ASD) (Table S11). A doubling in blood cadmium was marginally associated with 1.11 (95% CI 0.96, 1.29) times higher risk of ASD, and a doubling in blood lead was associated with 1.23 (95% CI 1.01, 1.54) times higher risk of ASD (Figure S4). A doubling in cadmium was also associated with 1.10 (95% CI 1.02, 1.19) times higher risk of non-typical development. A doubling in blood lead was associated with 1.16 (95% CI 1.00, 1.35) times higher risk of non-typical development (Figure S4). No associations were observed between the remaining blood metal concentrations (mercury, selenium, manganese) and neurodevelopmental status.

### Sensitivity analysis

With batch as a covariate (Tables S12 and S13), the cadmium association in T1/T2 pregnancy with ASD remained consistent where being over the limit of detection was associated with 1.68 (95% CI 1.08, 2.62) times higher risk of ASD. With batch adjustment, antimony in T1/T2 pregnancy was marginally associated with ASD, with estimated RR = 1.64 (95% CI 0.98, 2.73). The estimated associations between cesium and ASD had consistent magnitude with slight attenuation. On the other hand, the relationships with thallium and tin with ASD were attenuated.

Using logistic regression models, consistency to the previous log binomial findings was observed for cadmium and cesium. In general, estimates on the odds ratio scale were higher in magnitude and significance for cadmium and cesium. In particular, the T1/T2 cadmium association with non-typically developing was stronger in logistic regression, with an estimated OR = 1.95 (95% CI 1.05, 3.63). Strength of relationships between T1/T2 arsenic, thallium, and tin with ASD were attenuated with larger confidence intervals, while T3 arsenic showed marginal association with ASD (OR = 1.21, 95% CI 0.97, 1.49) when using logistic regression (Table S14)**.** T1/T2 pregnancy molybdenum and nickel associations with non-typical development strengthened in logistic regression, while selenium, zinc, and mercury associations with non-typical development were attenuated (Table S15)**.**

## Discussion

In two prospective birth cohorts of siblings of children with ASD, we measured urinary metals levels during two pregnancy time periods and examined relationships to ASD or non-typical development status at age 3 in an ExWAS framework. Our most consistent finding was heightened risk of atypical neurodevelopment related to T1/T2 cadmium exposure. Although the relationships were not significant in T3 pregnancy, the directions of effect were consistent across time periods. Furthermore, similar findings were observed in the blood subsample. Cesium related to atypical neurodevelopment was also notable, with consistency across ASD and non-typical development outcomes and time period, with exception of T3 pregnancy cesium and non-typical development. Cadmium and cesium associations were also the most robust to different modelling strategies. This study suggests metals exposure during pregnancy may be related to risk of ASD or non-typical development status at age 3. Existing studies have also examined the relationship between metals exposure and ASD with considerable heterogeneity in exposure timing and matrices measured [39].

Our findings with cadmium align with previous results, while cesium differed. The study in the Norwegian Mother, Father, and Child Cohort Study found higher odds of ASD for children in the highest quartile of cadmium exposure measured in blood during pregnancy [24], matching results from the present study. The same study found the highest quartile of pregnancy blood cesium levels had lower odds of ASD compared to the lowest quartile, while in contrast our study suggests higher risk of ASD with higher urinary cesium. Cadmium exposure can occur through release into the environment by industrial processes such as smelting and battery production, and subsequent bioaccumulation in plants, including tobacco [34]. Thus, diet and smoking may play a role in observed associations. Though our analysis was restricted to those who did not smoke during pregnancy, exposure through second-hand smoke is possible. Furthermore, cadmium exposure can occur though air pollution [34], and air pollution during pregnancy has been associated with ASD [40, 41]. Cadmium exposure is associated with adverse pregnancy and birth outcomes, and can accumulate in the placenta [42]. Possible mechanisms of health impacts of cadmium include impacting gap junction formation in embryo development, oxidative stress in the placenta, disruption of nutrient homeostasis in the placenta, and aberrant DNA methylation and subsequent gene expression [42, 43]. Cesium has been detected in ash from coal power plants and hazardous waste incineration, and can be absorbed in plants after deposition, with most human exposure occurring through diet [44]. Cesium has been related to birth outcomes such as small for gestational age [45]. Correlation between cesium and thallium may have contributed to observed associations between thallium and ASD, driven primarily by MARBLES. In MARBLES, thallium and cesium were correlated (Spearman *r* = 0.45), while in EARLI where thallium associations were not observed the two metals were only moderately correlated (Spearman *r* = 0.23). However, thallium has been found to at higher levels in urine of individuals with ASD compared to neurotypical controls [46], and has been related to pre-term birth with proposed mechanisms of toxicity including ability to cross placenta and oxidative stress [47].

Lead is a known neurotoxicant, and exposure matrix was an important factor. A systematic review and meta-analysis of lead concentrations in children with ASD from cross-sectional and case–control studies showed significant difference in child blood lead levels (samples collected in ASD and control group children at mean age 6.91 and 6.74 years respectively) compared to controls, but not in child urinary lead levels (samples collected in ASD and control group children at mean age 8.64 and 8.47 years respectively) [48]**.** This mirrors our results in measures during pregnancy, where we found blood lead levels of pregnant individuals were associated with risk of ASD or non-typical development in offspring, but not pregnancy urinary lead levels. Blood lead is a more reliable measure of recent exposure compared to urinary or hair lead levels [49], which may explain our findings of stronger blood lead ASD associations than those seen with urinary lead.

Our results for selenium and arsenic were mixed. A doubling of T3 pregnancy selenium concentration was associated with lower risk of non-typical development, however there were opposite effect estimates between cohorts. Selenium supplementation in an animal model attenuated autism phenotype [50], and studies measuring selenium cross-sectionally in children in Saudi Arabia [51] and China [52] found lower selenium levels in those with ASD. On the other hand, two-sample Mendelian randomization analysis using genetic instruments of blood and blood-toenail selenium suggest selenium levels are associated with increased risk of ASD [53], and in the Boston Birth Cohort red blood cell selenium levels measured during pregnancy at near delivery were associated with increased odds of ASD in children [54]. Selenium’s main route of exposure is through diet containing selenium rich foods or plants/animals that have bioaccumulated selenium from industrial or agricultural release [34]. Thus, findings and cohort differences may be due to uncorrected confounding from impact of diet during pregnancy. We found T1/T2 arsenic to be protective for ASD, but only in the EARLI cohort. In contrast, higher arsenic in blood during pregnancy has previously been seen to be associated with increased ASD [24]. Urinary arsenic measures both organic and inorganic arsenic, which can be impacted by seafood consumption [34]. Additionally, erythrocyte levels of arsenic have been seen to increase with fresh fruit consumption [55]. Urinary arsenic measures and associations in this and other studies may be confounded by dietary patterns, which may differ between cohorts.

Our findings add to a growing body of evidence of the neurodevelopmental impacts of metals exposure during pregnancy, and has several strengths. We were able to assay a wide array of metals with high detection rates in two different birth cohorts, at two different time periods. In one cohort, we were also able to evaluate five metals in a different exposure matrix: blood during pregnancy. The longitudinal design allowed examination of exposure measures during pregnancy that preceded subsequent ASD outcome three years after birth. The enriched risk cohort design ensured all participants were clinically assessed using gold standards for ASD diagnosis.

We used the ExWAS framework to consistently perform evaluations across metals, time periods, and cohorts. This approach has been effectively used with other outcomes to screen exposures for further testing with laboratory and population based approaches [25, 26]. Our hypothesis generating findings prioritize cadmium and cesium for examination with ASD. In the ExWAS approach, we considered each metal individually. This is complementary to mixtures approaches, which consider combinations of metals together [56]. Mixtures approaches offer important ways to account for correlations and interactions among exposures. In these same cohorts, mixtures of metals were tested with a continuous neurodevelopmental outcome measure using Bayesian kernel machine regression [57]. Combinations of exposures to lead, mercury, selenium and manganese had inconsistent associations with Social Responsiveness Score at age three across the EARLI and MARBLES cohorts [57]. ExWAS and mixtures approaches both provide useful and distinct information about the complex ways exposures can be related to health.

Our study has several limitations, which may point to areas of future research. This study modeled metals as linear or dichotomous, but some metals, especially essential nutrients, may have non-linear relationships. While the sibling cohort design allowed for an extensively phenotyped sample, our findings may not be generalizable to populations where ASD is less common, thus it would be important to also compare to results found in population-based samples. Genetic factors contributing to probability of ASD will likely be enriched in our study population, which recruited families who already had a child with ASD. Since disrupted elemental metabolism has been seen in relation to ASD [23], unaccounted genetic factors impacting metals metabolism may confound associations. Additionally, future studies should consider other exposure matrices or time periods. The choice of exposure matrix is important for exposure timing. For example, blood cadmium levels reflect recent exposure, while urinary cadmium reflects a longer, cumulative exposure [58]. Certain exposure matrices may be more reliable for some metals. Since urine samples for analysis were only available for a subset of cohort participants, selection bias may impact our results if availability or willingness to provide urine samples is related to behaviors associated with metals exposures. Our blood sample was further restricted, and selection bias may exist, especially if micro-clotting was associated with metals exposures. Future analyses in large and prospective cohorts may increase the generalizability of results.

## Conclusion

This study suggests that prenatal exposure to toxic metals, such as cadmium, is associated with risk of ASD or non-typical development in offspring. Potential routes of exposure to metals include contamination of soil and water, through ambient air, and through use in industrial applications or domestic products [59]. Further studies determining the population attributable risk from metals exposures and establishment of causality are necessary to evaluate whether public health measures to reduce these exposures during pregnancy can be used as preventative strategy for neurodevelopmental disorders.

### Supplementary Information

Supplementary Material 1.

## Data Availability

Data used in this manuscript is publicly available through the National Institute of Mental Health Data Archive (EARLI cohort repository: 1600, MARBLES cohort repository: 1946, EARLI/MARBLES metals repository: 2462) and through data requests to the Principal Investigators of cohorts (EARLI: MDF, MARBLES: RJS).

## Abbreviations

ASD: Autism spectrum disorder
EARLI: Early autism risk longitudinal investigation
MARBLES: Markers of autism risk in babies - learning early signs
ADOS: Autism diagnostic observation schedule
MSEL: Mullen scales of early learning
LOD: Limit of detection
T1: Trimester 1
T2: Trimester 2
T3: Trimester 3
ExWAS: Exposure-wide association study
