# Supplementary material for: Exposure to heavy metals in utero and autism spectrum disorder at age 3: a meta-analysis of two longitudinal cohorts of siblings of children with autism
Source: Environ Health. 2024 Jul 5;23:62. doi: 10.1186/s12940-024-01101-2 (PMC11225197; doi:10.1186/s12940-024-01101-2)
Supplement: Supplementary file 1 — Supplementary Material 1. [file 12940_2024_1101_MOESM1_ESM.docx]

**Title:** Exposure to heavy metals in utero and autism spectrum disorder at age 3: A meta-analysis of two longitudinal cohorts of siblings of children with autism

**Supplementary Information**

This supplementary information contains 15 supplemental tables and 4 supplemental figures.

**Table S1.** Timing of exposure reflected in urinary metals measures.

| **Metal** | **Urinary Measures Timing** | **Source** |
| --- | --- | --- |
| Antimony | recent exposure, half-life 95 hrs | https://www.cdc.gov/biomonitoring/Antimony_BiomonitoringSummary.html |
| Arsenic | recent exposure, half-life 2-4 days | https://www.cdc.gov/biomonitoring/Arsenic_BiomonitoringSummary.html |
| Barium | recent exposure, half-life ~3 days | https://www.cdc.gov/biomonitoring/Barium_BiomonitoringSummary.html |
| Beryllium | recent and accumulated exposure | https://www.cdc.gov/biomonitoring/Beryllium_BiomonitoringSummary.html |
| Cadmium | cumulative exposure, half-life 1-4 decades | https://www.cdc.gov/biomonitoring/Cadmium_BiomonitoringSummary.html |
| Cesium | recent exposure, half-life 70-109 days | https://www.cdc.gov/biomonitoring/Cesium_BiomonitoringSummary.html |
| Chromium | recent exposure, chromium(III) half-life 10–40 hours, chromium(VI) half life ~25–35 days | https://www.atsdr.cdc.gov/toxprofiles/tp7.pdf |
| Cobalt | recent exposure, occupational exposures have produced elevated levels weeks | https://www.cdc.gov/biomonitoring/Cobalt_BiomonitoringSummary.html |
| Copper | recent exposure, but may also reflect disruption of homeostasis and liver | https://www.atsdr.cdc.gov/ToxProfiles/tp132.pdf |
| Lead | recent exposure, though there is greater individual variation in urine lead than in blood and greater potential for contamination | https://www.cdc.gov/biomonitoring/Lead_BiomonitoringSummary.html |
| Manganese | unclear, measurements of manganese may only be related to exposure after exposure has stopped, also excess manganese is primarily excreted in bile, not urine | https://www.atsdr.cdc.gov/toxprofiles/tp151.pdf |
| Mercury | kinetics of different forms vary, half-life 1-3 months | https://www.cdc.gov/biomonitoring/Mercury_BiomonitoringSummary.html |
| Molybdenum | recent exposure, reflects dietary intake | https://www.atsdr.cdc.gov/ToxProfiles/tp212.pdf |
| Nickel | recent exposure, half-life 28 hours | https://www.atsdr.cdc.gov/toxguides/toxguide-15.pdf |
| Platinum | recent exposure | https://www.cdc.gov/biomonitoring/Platinum_BiomonitoringSummary.html |
| Selenium | recent exposure | Martinez-Morata I, Sobel M, Tellez-Plaza M, Navas-Acien A, Howe CG, Sanchez TR. A State-of-the-Science Review on Metal Biomarkers. Curr Environ Health Rep. 2023 Sep;10(3):215-249. |
| Thallium | recent exposure | https://www.cdc.gov/biomonitoring/Thallium_BiomonitoringSummary.html |
| Tin | cumulative exposure, half-life 1-3 days in soft tissues, 3-4 months in bone in animal models | https://www.atsdr.cdc.gov/ToxProfiles/tp55.pdf |
| Tungsten | recent exposure | https://www.cdc.gov/biomonitoring/Tungsten_BiomonitoringSummary.html |
| Uranium | half-life 24 hrs-15 days, half-life of insoluble uranium several years in lung | https://www.cdc.gov/biomonitoring/Uranium_BiomonitoringSummary.html |
| Vanadium | recent exposure, half-life 20-40 hours in urine | Barceloux DG. Vanadium. J Toxicol Clin Toxicol. 1999;37(2):265-78. doi: 10.1081/clt-100102425. Erratum in: J Toxicol Clin Toxicol 2000;38(7):813. |
| Zinc | recent exposure | Martinez-Morata I, Sobel M, Tellez-Plaza M, Navas-Acien A, Howe CG, Sanchez TR. A State-of-the-Science Review on Metal Biomarkers. Curr Environ Health Rep. 2023 Sep;10(3):215-249. doi: 10.1007/s40572-023-00402-x. Epub 2023 Jun 20. |

**Table S2**. Number of samples by cohort, pregnancy timepoint, and batches having different limits of detection.

| **Metal** | **Cohort** | **LOD** | **Time Period** | **N** | **N above LOD** | **% over LOD** |
| --- | --- | --- | --- | --- | --- | --- |
| Antimony | EARLI | 0.04 | T1/T2 | 37 | 23 | 62.2 |
| Antimony | EARLI | 0.04 | T3 | 37 | 21 | 56.8 |
| Antimony | EARLI | 0.08 | T1/T2 | 117 | 17 | 14.5 |
| Antimony | EARLI | 0.08 | T3 | 120 | 17 | 14.2 |
| Antimony | MARBLES | 0.04 | T1/T2 | 9 | 4 | 44.4 |
| Antimony | MARBLES | 0.04 | T3 | 26 | 8 | 30.8 |
| Antimony | MARBLES | 0.08 | T1/T2 | 151 | 21 | 13.9 |
| Antimony | MARBLES | 0.08 | T3 | 203 | 25 | 12.3 |
| Arsenic | EARLI | 0.3 | T1/T2 | 36 | 36 | 100 |
| Arsenic | EARLI | 0.3 | T3 | 36 | 36 | 100 |
| Arsenic | EARLI | 0.6 | T1/T2 | 117 | 116 | 99.1 |
| Arsenic | EARLI | 0.6 | T3 | 121 | 121 | 100 |
| Arsenic | EARLI | 3 | T1/T2 | 1 | 1 | 100 |
| Arsenic | MARBLES | 0.3 | T1/T2 | 23 | 23 | 100 |
| Arsenic | MARBLES | 0.3 | T3 | 46 | 46 | 100 |
| Arsenic | MARBLES | 0.6 | T1/T2 | 137 | 137 | 100 |
| Arsenic | MARBLES | 0.6 | T3 | 183 | 182 | 99.5 |
| Barium | EARLI | 0.1 | T1/T2 | 37 | 37 | 100 |
| Barium | EARLI | 0.1 | T3 | 37 | 35 | 94.6 |
| Barium | EARLI | 0.2 | T1/T2 | 117 | 115 | 98.3 |
| Barium | EARLI | 0.2 | T3 | 120 | 118 | 98.3 |
| Barium | MARBLES | 0.1 | T1/T2 | 9 | 9 | 100 |
| Barium | MARBLES | 0.1 | T3 | 26 | 26 | 100 |
| Barium | MARBLES | 0.2 | T1/T2 | 151 | 148 | 98 |
| Barium | MARBLES | 0.2 | T3 | 203 | 202 | 99.5 |
| Cadmium | EARLI | 0.06 | T1/T2 | 37 | 23 | 62.2 |
| Cadmium | EARLI | 0.06 | T3 | 36 | 22 | 61.1 |
| Cadmium | EARLI | 0.12 | T1/T2 | 117 | 58 | 49.6 |
| Cadmium | EARLI | 0.12 | T3 | 121 | 50 | 41.3 |
| Cadmium | MARBLES | 0.06 | T1/T2 | 23 | 8 | 34.8 |
| Cadmium | MARBLES | 0.06 | T3 | 46 | 9 | 19.6 |
| Cadmium | MARBLES | 0.12 | T1/T2 | 137 | 47 | 34.3 |
| Cadmium | MARBLES | 0.12 | T3 | 183 | 51 | 27.9 |
| Cesium | EARLI | 0.1 | T1/T2 | 37 | 37 | 100 |
| Cesium | EARLI | 0.1 | T3 | 37 | 36 | 97.3 |
| Cesium | EARLI | 0.2 | T1/T2 | 117 | 117 | 100 |
| Cesium | EARLI | 0.2 | T3 | 120 | 120 | 100 |
| Cesium | MARBLES | 0.1 | T1/T2 | 9 | 9 | 100 |
| Cesium | MARBLES | 0.1 | T3 | 26 | 26 | 100 |
| Cesium | MARBLES | 0.2 | T1/T2 | 151 | 151 | 100 |
| Cesium | MARBLES | 0.2 | T3 | 203 | 203 | 100 |
| Chromium | EARLI | 0.4 | T1/T2 | 37 | 7 | 18.9 |
| Chromium | EARLI | 0.4 | T3 | 36 | 7 | 19.4 |
| Chromium | EARLI | 0.8 | T1/T2 | 117 | 38 | 32.5 |
| Chromium | EARLI | 0.8 | T3 | 121 | 22 | 18.2 |
| Chromium | MARBLES | 0.4 | T1/T2 | 23 | 1 | 4.3 |
| Chromium | MARBLES | 0.4 | T3 | 46 | 4 | 8.7 |
| Chromium | MARBLES | 0.8 | T1/T2 | 137 | 28 | 20.4 |
| Chromium | MARBLES | 0.8 | T3 | 183 | 26 | 14.2 |
| Cobalt | EARLI | 0.05 | T1/T2 | 37 | 37 | 100 |
| Cobalt | EARLI | 0.05 | T3 | 37 | 36 | 97.3 |
| Cobalt | EARLI | 0.1 | T1/T2 | 117 | 117 | 100 |
| Cobalt | EARLI | 0.1 | T3 | 120 | 120 | 100 |
| Cobalt | MARBLES | 0.05 | T1/T2 | 9 | 9 | 100 |
| Cobalt | MARBLES | 0.05 | T3 | 26 | 26 | 100 |
| Cobalt | MARBLES | 0.1 | T1/T2 | 151 | 150 | 99.3 |
| Cobalt | MARBLES | 0.1 | T3 | 203 | 203 | 100 |
| Copper | EARLI | 2.5 | T1/T2 | 37 | 35 | 94.6 |
| Copper | EARLI | 2.5 | T3 | 37 | 36 | 97.3 |
| Copper | EARLI | 5 | T1/T2 | 117 | 107 | 91.5 |
| Copper | EARLI | 5 | T3 | 120 | 113 | 94.2 |
| Copper | MARBLES | 2.5 | T1/T2 | 9 | 9 | 100 |
| Copper | MARBLES | 2.5 | T3 | 26 | 26 | 100 |
| Copper | MARBLES | 5 | T1/T2 | 151 | 127 | 84.1 |
| Copper | MARBLES | 5 | T3 | 203 | 170 | 83.7 |
| Lead | EARLI | 0.1 | T1/T2 | 37 | 20 | 54.1 |
| Lead | EARLI | 0.1 | T3 | 37 | 20 | 54.1 |
| Lead | EARLI | 0.2 | T1/T2 | 117 | 76 | 65 |
| Lead | EARLI | 0.2 | T3 | 120 | 79 | 65.8 |
| Lead | MARBLES | 0.1 | T1/T2 | 9 | 2 | 22.2 |
| Lead | MARBLES | 0.1 | T3 | 26 | 7 | 26.9 |
| Lead | MARBLES | 0.2 | T1/T2 | 151 | 63 | 41.7 |
| Lead | MARBLES | 0.2 | T3 | 203 | 87 | 42.9 |
| Manganese | EARLI | 0.08 | T1/T2 | 37 | 26 | 70.3 |
| Manganese | EARLI | 0.08 | T3 | 37 | 19 | 51.4 |
| Manganese | EARLI | 0.16 | T1/T2 | 117 | 93 | 79.5 |
| Manganese | EARLI | 0.16 | T3 | 120 | 99 | 82.5 |
| Manganese | MARBLES | 0.08 | T1/T2 | 9 | 9 | 100 |
| Manganese | MARBLES | 0.08 | T3 | 26 | 24 | 92.3 |
| Manganese | MARBLES | 0.16 | T1/T2 | 151 | 117 | 77.5 |
| Manganese | MARBLES | 0.16 | T3 | 203 | 143 | 70.4 |
| Mercury | EARLI | 0.05 | T1/T2 | 37 | 33 | 89.2 |
| Mercury | EARLI | 0.05 | T3 | 37 | 31 | 83.8 |
| Mercury | EARLI | 0.1 | T1/T2 | 117 | 105 | 89.7 |
| Mercury | EARLI | 0.1 | T3 | 120 | 101 | 84.2 |
| Mercury | MARBLES | 0.05 | T1/T2 | 9 | 8 | 88.9 |
| Mercury | MARBLES | 0.05 | T3 | 26 | 22 | 84.6 |
| Mercury | MARBLES | 0.1 | T1/T2 | 151 | 115 | 76.2 |
| Mercury | MARBLES | 0.1 | T3 | 203 | 121 | 59.6 |
| Molybdenum | EARLI | 0.3 | T1/T2 | 36 | 36 | 100 |
| Molybdenum | EARLI | 0.3 | T3 | 36 | 36 | 100 |
| Molybdenum | EARLI | 0.6 | T1/T2 | 118 | 118 | 100 |
| Molybdenum | EARLI | 0.6 | T3 | 121 | 121 | 100 |
| Molybdenum | MARBLES | 0.3 | T1/T2 | 23 | 23 | 100 |
| Molybdenum | MARBLES | 0.3 | T3 | 46 | 46 | 100 |
| Molybdenum | MARBLES | 0.6 | T1/T2 | 137 | 137 | 100 |
| Molybdenum | MARBLES | 0.6 | T3 | 183 | 183 | 100 |
| Nickel | EARLI | 0.8 | T1/T2 | 37 | 36 | 97.3 |
| Nickel | EARLI | 0.8 | T3 | 37 | 36 | 97.3 |
| Nickel | EARLI | 1.6 | T1/T2 | 117 | 93 | 79.5 |
| Nickel | EARLI | 1.6 | T3 | 120 | 103 | 85.8 |
| Nickel | MARBLES | 0.8 | T1/T2 | 9 | 9 | 100 |
| Nickel | MARBLES | 0.8 | T3 | 26 | 26 | 100 |
| Nickel | MARBLES | 1.6 | T1/T2 | 151 | 145 | 96 |
| Nickel | MARBLES | 1.6 | T3 | 203 | 191 | 94.1 |
| Selenium | EARLI | 2 | T1/T2 | 37 | 37 | 100 |
| Selenium | EARLI | 2 | T3 | 36 | 36 | 100 |
| Selenium | EARLI | 4 | T1/T2 | 117 | 117 | 100 |
| Selenium | EARLI | 4 | T3 | 121 | 121 | 100 |
| Selenium | MARBLES | 2 | T1/T2 | 23 | 23 | 100 |
| Selenium | MARBLES | 2 | T3 | 46 | 46 | 100 |
| Selenium | MARBLES | 4 | T1/T2 | 137 | 137 | 100 |
| Selenium | MARBLES | 4 | T3 | 183 | 183 | 100 |
| Thallium | EARLI | 0.02 | T1/T2 | 37 | 37 | 100 |
| Thallium | EARLI | 0.02 | T3 | 37 | 36 | 97.3 |
| Thallium | EARLI | 0.04 | T1/T2 | 117 | 115 | 98.3 |
| Thallium | EARLI | 0.04 | T3 | 120 | 117 | 97.5 |
| Thallium | MARBLES | 0.02 | T1/T2 | 9 | 9 | 100 |
| Thallium | MARBLES | 0.02 | T3 | 26 | 24 | 92.3 |
| Thallium | MARBLES | 0.04 | T1/T2 | 151 | 143 | 94.7 |
| Thallium | MARBLES | 0.04 | T3 | 203 | 190 | 93.6 |
| Tin | EARLI | 0.1 | T1/T2 | 37 | 34 | 91.9 |
| Tin | EARLI | 0.1 | T3 | 37 | 25 | 67.6 |
| Tin | EARLI | 0.2 | T1/T2 | 117 | 85 | 72.6 |
| Tin | EARLI | 0.2 | T3 | 120 | 86 | 71.7 |
| Tin | MARBLES | 0.1 | T1/T2 | 9 | 7 | 77.8 |
| Tin | MARBLES | 0.1 | T3 | 26 | 25 | 96.2 |
| Tin | MARBLES | 0.2 | T1/T2 | 151 | 117 | 77.5 |
| Tin | MARBLES | 0.2 | T3 | 203 | 140 | 69 |
| Zinc | EARLI | 2 | T1/T2 | 37 | 37 | 100 |
| Zinc | EARLI | 2 | T3 | 37 | 36 | 97.3 |
| Zinc | EARLI | 4 | T1/T2 | 117 | 117 | 100 |
| Zinc | EARLI | 4 | T3 | 120 | 120 | 100 |
| Zinc | MARBLES | 2 | T1/T2 | 9 | 9 | 100 |
| Zinc | MARBLES | 2 | T3 | 26 | 26 | 100 |
| Zinc | MARBLES | 4 | T1/T2 | 151 | 151 | 100 |
| Zinc | MARBLES | 4 | T3 | 203 | 203 | 100 |

**Table S3**. Number of samples dropped from analysis based upon being an outlier > 5 standard deviations from the mean in log transformed measures.

| **Metal** | **EARLI Trimester 1/ Trimester 2** | **EARLI Trimester 3** | **MARBLES Trimester 1/ Trimester 2** | **MARBLES Trimester 3** |
| --- | --- | --- | --- | --- |
| Antimony | 0 | 0 | 0 | 0 |
| Arsenic | 0 | 0 | 0 | 0 |
| Barium | 0 | 0 | 0 | 0 |
| Cadmium | 0 | 0 | 0 | 0 |
| Cesium | 0 | 0 | 0 | 1 |
| Chromium | 0 | 0 | 0 | 0 |
| Cobalt | 0 | 0 | 0 | 0 |
| Copper | 0 | 1 | 0 | 2 |
| Lead | 0 | 0 | 0 | 0 |
| Manganese | 0 | 0 | 0 | 1 |
| Mercury | 0 | 0 | 0 | 0 |
| Molybdenum | 0 | 0 | 0 | 0 |
| Nickel | 0 | 0 | 0 | 0 |
| Selenium | 0 | 0 | 0 | 0 |
| Thallium | 0 | 0 | 0 | 0 |
| Tin | 0 | 0 | 0 | 0 |
| Zinc | 0 | 0 | 0 | 0 |

**Table S4.** Distribution of raw measures^1^ of metals concentrations in trimester 1/trimester 2 pregnancy urine samples by cohort. All metals concentrations are measured in parts per billion (ppb). Beryllium, platinum, tungsten, uranium, and vanadium were dropped from analysis.

| **Metal** | **Cohort** | **Mean (ppb)** | **Standard deviation** | **Median (ppb)** | **Interquartile range** | **N** | **N Above LOD** | **Percent Above LOD** |
| --- | --- | --- | --- | --- | --- | --- | --- | --- |
| Antimony | EARLI | 0.0486 | 0.0545 | 0.0383 | 0.0246 | 154 | 40 | 26 |
|  | MARBLES | 0.0199 | 0.0988 | 0.0353 | 0.05 | 160 | 25 | 15.6 |
| Arsenic | EARLI | 23.3 | 96.7 | 6.76 | 9.74 | 154 | 153 | 99.4 |
|  | MARBLES | 11 | 17.2 | 6.5 | 6.01 | 160 | 160 | 100 |
| Barium | EARLI | 2.38 | 2.25 | 1.77 | 2.27 | 154 | 152 | 98.7 |
|  | MARBLES | 2.43 | 1.89 | 1.86 | 2.14 | 160 | 157 | 98.1 |
| Beryllium | EARLI | 0.00604 | 0.0708 | -0.00607 | 0.0491 | 154 | 17 | 11 |
|  | MARBLES | 0.00131 | 0.0943 | 0.00239 | 0.0506 | 160 | 7 | 4.4 |
| Cadmium | EARLI | 0.115 | 0.128 | 0.109 | 0.133 | 154 | 81 | 52.6 |
|  | MARBLES | 0.104 | 0.125 | 0.0853 | 0.108 | 160 | 55 | 34.4 |
| Cesium | EARLI | 3.77 | 1.44 | 3.48 | 1.57 | 154 | 154 | 100 |
|  | MARBLES | 4.84 | 1.77 | 4.43 | 1.7 | 160 | 160 | 100 |
| Chromium | EARLI | 0.39 | 0.57 | 0.138 | 0.676 | 154 | 45 | 29.2 |
|  | MARBLES | 0.556 | 0.673 | 0.414 | 0.62 | 160 | 29 | 18.1 |
| Cobalt | EARLI | 0.693 | 0.404 | 0.591 | 0.461 | 154 | 154 | 100 |
|  | MARBLES | 0.98 | 0.495 | 0.879 | 0.543 | 160 | 159 | 99.4 |
| Copper | EARLI | 8.83 | 5.86 | 8.14 | 3.73 | 154 | 142 | 92.2 |
|  | MARBLES | 9.53 | 4.59 | 8.46 | 4.85 | 160 | 136 | 85 |
| Lead | EARLI | 0.251 | 0.245 | 0.21 | 0.232 | 154 | 96 | 62.3 |
|  | MARBLES | 0.224 | 0.243 | 0.16 | 0.192 | 160 | 65 | 40.6 |
| Manganese | EARLI | 0.252 | 0.221 | 0.233 | 0.197 | 154 | 119 | 77.3 |
|  | MARBLES | 0.335 | 0.315 | 0.262 | 0.201 | 160 | 126 | 78.8 |
| Mercury | EARLI | 0.318 | 0.338 | 0.202 | 0.274 | 154 | 138 | 89.6 |
|  | MARBLES | 0.309 | 0.595 | 0.175 | 0.279 | 160 | 123 | 76.9 |
| Molybdenum | EARLI | 60.3 | 40.4 | 48.3 | 39.9 | 154 | 154 | 100 |
|  | MARBLES | 62.6 | 33.6 | 53.7 | 37.3 | 160 | 160 | 100 |
| Nickel | EARLI | 3.44 | 2.34 | 3.01 | 2.55 | 154 | 129 | 83.8 |
|  | MARBLES | 5.85 | 2.12 | 5.56 | 2.7 | 160 | 154 | 96.2 |
| Platinum | EARLI | -0.0198 | 0.0449 | -0.013 | 0.0466 | 154 | 4 | 2.6 |
|  | MARBLES | 0.00893 | 0.0228 | 0.00606 | 0.0197 | 160 | 3 | 1.9 |
| Selenium | EARLI | 41.7 | 23.3 | 36.1 | 16.7 | 154 | 154 | 100 |
|  | MARBLES | 43.1 | 22.5 | 37.6 | 19.6 | 160 | 160 | 100 |
| Thallium | EARLI | 0.13 | 0.0726 | 0.118 | 0.0629 | 154 | 152 | 98.7 |
|  | MARBLES | 0.139 | 0.0809 | 0.121 | 0.0796 | 160 | 152 | 95 |
| Tin | EARLI | 0.555 | 0.831 | 0.324 | 0.401 | 154 | 119 | 77.3 |
|  | MARBLES | 0.661 | 1.13 | 0.353 | 0.433 | 160 | 124 | 77.5 |
| Tungsten | EARLI | 0.202 | 1.05 | 0.0842 | 0.101 | 154 | 15 | 9.7 |
|  | MARBLES | 0.209 | 0.669 | 0.112 | 0.153 | 160 | 22 | 13.8 |
| Uranium | EARLI | -0.00961 | 0.0185 | -0.00501 | 0.0199 | 154 | 7 | 4.5 |
|  | MARBLES | -0.0016 | 0.0286 | 0.000397 | 0.0129 | 160 | 14 | 8.8 |
| Vanadium | EARLI | 0.223 | 0.288 | 0.161 | 0.188 | 154 | 6 | 3.9 |
|  | MARBLES | 0.343 | 0.361 | 0.267 | 0.186 | 160 | 5 | 3.1 |
| Zinc | EARLI | 261 | 164 | 222 | 201 | 154 | 154 | 100 |
|  | MARBLES | 282 | 224 | 232 | 209 | 160 | 160 | 100 |

*Acronyms: Early Autism Risk Longitudinal Investigation (EARLI), Markers of Autism Risk in Babies-Learning Early Signs (MARBLES), limit of detection (LOD)*

*^1^Measurements depend on standard curves for calibration, and it is possible to obtain negative values for measurements, reflecting imprecision at the low exposure range. Measures below LOD were imputed before association tests.*

**Table S5.** Distribution of raw measures*^1^* of metals concentrations in trimester 3 pregnancy urine samples by cohort. All metals concentrations are measured in parts per billion (ppb). Beryllium, platinum, tungsten, uranium, and vanadium were dropped from analysis.

| **Metal** | **Cohort** | **Mean** | **Standard deviation** | **Median** | **Interquartile range** | **N** | **N Above LOD** | **Percent Above LOD** |
| --- | --- | --- | --- | --- | --- | --- | --- | --- |
| Antimony | EARLI | 0.0413 | 0.0237 | 0.0381 | 0.03 | 157 | 38 | 24.2 |
|  | MARBLES | 0.04 | 0.13 | 0.0337 | 0.05 | 231 | 34 | 14.7 |
| Arsenic | EARLI | 14.2 | 26.2 | 5.23 | 8.16 | 157 | 157 | 100 |
|  | MARBLES | 12.1 | 18.2 | 6.72 | 8.98 | 231 | 230 | 99.6 |
| Barium | EARLI | 3.41 | 2.96 | 2.58 | 2.94 | 157 | 153 | 97.5 |
|  | MARBLES | 3.88 | 4.26 | 2.67 | 3.37 | 231 | 230 | 99.6 |
| Beryllium | EARLI | 0.0134 | 0.0785 | 0.00187 | 0.042 | 157 | 16 | 10.2 |
|  | MARBLES | -0.00349 | 0.0643 | -0.00734 | 0.0528 | 231 | 11 | 4.8 |
| Cadmium | EARLI | 0.102 | 0.165 | 0.0861 | 0.127 | 157 | 72 | 45.9 |
|  | MARBLES | 0.0698 | 0.106 | 0.0634 | 0.108 | 231 | 60 | 26 |
| Cesium | EARLI | 3.42 | 1.6 | 3.1 | 1.55 | 157 | 156 | 99.4 |
|  | MARBLES | 4.74 | 2.93 | 4.26 | 2.13 | 231 | 231 | 100 |
| Chromium | EARLI | 0.317 | 0.619 | 0.0984 | 0.291 | 157 | 29 | 18.5 |
|  | MARBLES | 0.503 | 0.63 | 0.287 | 0.596 | 231 | 31 | 13.4 |
| Cobalt | EARLI | 1.14 | 0.624 | 1.07 | 0.683 | 157 | 156 | 99.4 |
|  | MARBLES | 1.51 | 0.782 | 1.36 | 0.841 | 231 | 231 | 100 |
| Copper | EARLI | 11.9 | 12.4 | 9.82 | 5.4 | 157 | 149 | 94.9 |
|  | MARBLES | 15.8 | 34.7 | 11.2 | 7.88 | 231 | 197 | 85.3 |
| Lead | EARLI | 0.278 | 0.425 | 0.197 | 0.244 | 157 | 99 | 63.1 |
|  | MARBLES | 0.302 | 0.607 | 0.174 | 0.238 | 231 | 95 | 41.1 |
| Manganese | EARLI | 0.305 | 0.268 | 0.239 | 0.265 | 157 | 118 | 75.2 |
|  | MARBLES | 0.429 | 0.929 | 0.264 | 0.258 | 231 | 169 | 73.2 |
| Mercury | EARLI | 0.228 | 0.213 | 0.163 | 0.172 | 157 | 132 | 84.1 |
|  | MARBLES | 0.272 | 0.668 | 0.14 | 0.208 | 231 | 144 | 62.3 |
| Molybdenum | EARLI | 52.2 | 43.3 | 41.9 | 29.2 | 157 | 157 | 100 |
|  | MARBLES | 63.5 | 44.8 | 50 | 45.6 | 231 | 231 | 100 |
| Nickel | EARLI | 3.96 | 2.19 | 3.43 | 2.45 | 157 | 139 | 88.5 |
|  | MARBLES | 6.42 | 2.98 | 5.85 | 3.96 | 231 | 219 | 94.8 |
| Platinum | EARLI | -0.0131 | 0.0467 | -0.00458 | 0.0319 | 157 | 2 | 1.3 |
|  | MARBLES | 0.0059 | 0.0249 | 0.00297 | 0.021 | 231 | 1 | 0.4 |
| Selenium | EARLI | 36 | 14.3 | 32.8 | 13.2 | 157 | 157 | 100 |
|  | MARBLES | 38 | 15 | 36.7 | 19.1 | 231 | 231 | 100 |
| Thallium | EARLI | 0.134 | 0.0817 | 0.117 | 0.0772 | 157 | 153 | 97.5 |
|  | MARBLES | 0.123 | 0.0661 | 0.114 | 0.0796 | 231 | 216 | 93.5 |
| Tin | EARLI | 0.706 | 1.84 | 0.303 | 0.389 | 157 | 111 | 70.7 |
|  | MARBLES | 0.761 | 1.59 | 0.357 | 0.516 | 231 | 167 | 72.3 |
| Tungsten | EARLI | 0.0842 | 0.112 | 0.0516 | 0.0791 | 157 | 7 | 4.5 |
|  | MARBLES | 0.169 | 0.439 | 0.105 | 0.153 | 231 | 26 | 11.3 |
| Uranium | EARLI | -0.00865 | 0.0227 | -0.00279 | 0.0182 | 157 | 10 | 6.4 |
|  | MARBLES | 0.00046 | 0.0239 | -0.00096 | 0.0135 | 231 | 25 | 10.8 |
| Vanadium | EARLI | 0.24 | 0.25 | 0.17 | 0.214 | 157 | 12 | 7.6 |
|  | MARBLES | 0.352 | 0.382 | 0.276 | 0.205 | 231 | 5 | 2.2 |
| Zinc | EARLI | 332 | 207 | 295 | 306 | 157 | 156 | 99.4 |
|  | MARBLES | 329 | 222 | 295 | 257 | 231 | 231 | 100 |

*Acronyms: Early Autism Risk Longitudinal Investigation (EARLI), Markers of Autism Risk in Babies-Learning Early Signs (MARBLES), limit of detection (LOD)*

*^1^Measurements depend on standard curves for calibration, and it is possible to obtain negative values for measurements, reflecting imprecision at the low exposure range. Measures below LOD were imputed before association tests.*

**Table S6.** Pregnant person and child characteristics of participants in the analytic sample with measures of metal exposure in urine from trimester 1 or trimester 2 (T1/T2) of pregnancy (<28 weeks of gestation). Data are split by cohort and comparted by neurodevelopmental status of the sibling. Distributions of categorical variables are compared with a chi-square test and continuous variables are compared with ANOVA test.

| **EARLI cohort** | **Typically developing** | **Non-typically developing** | **Autism spectrum disorder** | **P-value** |
| --- | --- | --- | --- | --- |
|  | ***N = 63*** | ***N = 67*** | ***N = 21*** |  |
| Education |  |  |  | 0.010 |
| College Degree | 46 (73%) | 37 (55%) | 8 (38%) |  |
| No Degree | 17 (27%) | 30 (45%) | 13 (62%) |  |
| Age | 35.08 (4.5) | 33.25 (4.7) | 33.52 (4.0) | 0.13 |
| Self Report Race/Ethnicity |  |  |  | 0.11 |
| Asian or Pacific Islander | 9 (14%) | 12 (18%) | 3 (14%) |  |
| Black | 4 (6.3%) | 9 (13%) | 0 (0%) |  |
| Hispanic | 6 (9.5%) | 11 (16%) | 6 (29%) |  |
| Other/Multiracial | 2 (3.2%) | 6 (9.0%) | 1 (4.8%) |  |
| White | 42 (67%) | 29 (43%) | 11 (52%) |  |
| Infant Sex |  |  |  | 0.006 |
| Female | 37 (59%) | 35 (52%) | 4 (19%) |  |
| Male | 26 (41%) | 32 (48%) | 17 (81%) |  |
| Gestational Age at Sample Collection (weeks) | 18.69 (4.8) | 17.83 (5.8) | 16.98 (7.1) | >0.9 |
| **MARBLES cohort** | **Typically developing** | **Non-typically developing** | **Autism spectrum disorder** | **P-value** |
|  | ***N=100*** | ***N=17*** | ***N=34*** |  |
| Education |  |  |  | 0.4 |
| College Degree | 60 (60%) | 9 (53%) | 16 (47%) |  |
| No Degree | 40 (40%) | 8 (47%) | 18 (53%) |  |
| Age | 34.60 (4.7) | 34.47 (4.0) | 34.96 (4.9) | 0.8 |
| Self Report Race/Ethnicity |  |  |  | 0.4 |
| Asian or Pacific Islander | 15 (15%) | 3 (18%) | 4 (12%) |  |
| Black | 2 (2.0%) | 2 (12%) | 3 (8.8%) |  |
| Hispanic | 23 (23%) | 3 (18%) | 5 (15%) |  |
| Other/Multiracial | 2 (2.0%) | 0 (0%) | 2 (5.9%) |  |
| White | 58 (58%) | 9 (53%) | 20 (59%) |  |
| Infant Sex |  |  |  | 0.076 |
| Female | 48 (48%) | 6 (35%) | 9 (26%) |  |
| Male | 52 (52%) | 11 (65%) | 25 (74%) |  |
| Gestational Age at Sample Collection (weeks) | 19.36 (4.0) | 18.86 (4.1) | 19.06 (4.0) | 0.8 |

*Acronyms: Early Autism Risk Longitudinal Investigation (EARLI), Markers of Autism Risk in Babies-Learning Early Signs (MARBLES)*

**Table S7.** Pregnant person and child characteristics of participants in the sample with measures of metal exposure in urine from trimester 3 (T3) of pregnancy (> 28 weeks of gestation). Data are split by cohort and comparted by neurodevelopmental status of the sibling. Distributions of categorical variables are compared with a chi-square test and continuous variables are compared with ANOVA test.

| **EARLI cohort** | **Typically developing** | **Non-typically developing** | **Autism spectrum disorder** | **P-value** |
| --- | --- | --- | --- | --- |
|  | ***N = 63*** | ***N = 68*** | ***N = 25*** |  |
| Education |  |  |  | 0.019 |
| College Degree | 45 (71%) | 35 (51%) | 11 (44%) |  |
| No Degree | 18 (29%) | 33 (49%) | 14 (56%) |  |
| Age | 34.98 (4.8) | 32.74 (4.7) | 33.92 (3.8) | 0.068 |
| Self Report Race/Ethnicity |  |  |  | 0.2 |
| Asian or Pacific Islander | 9 (14%) | 11 (16%) | 3 (12%) |  |
| Black | 2 (3.2%) | 11 (16%) | 3 (12%) |  |
| Hispanic | 7 (11%) | 11 (16%) | 4 (16%) |  |
| Other/Multiracial | 2 (3.2%) | 5 (7.4%) | 2 (8.0%) |  |
| White | 43 (68%) | 30 (44%) | 13 (52%) |  |
| Infant Sex |  |  |  | 0.004 |
| Female | 37 (59%) | 35 (51%) | 5 (20%) |  |
| Male | 26 (41%) | 33 (49%) | 20 (80%) |  |
| Gestational Age at Sample Collection (weeks) | 33.07 (2.9) | 33.13 (2.7) | 33.44 (3.3) | >0.9 |
| **MARBLES cohort** | **Typically developing** | **Non-typically developing** | **Autism spectrum disorder** | **P-value** |
|  | ***N=141*** | ***N=33*** | ***N=48*** |  |
| Education |  |  |  | 0.2 |
| College Degree | 79 (56%) | 15 (45%) | 21 (44%) |  |
| No Degree | 62 (44%) | 18 (55%) | 27 (56%) |  |
| Age | 34.03 (4.7) | 34.01 (4.4) | 34.80 (5.2) | 0.6 |
| Self Report Race/Ethnicity |  |  |  | 0.4 |
| Asian or Pacific Islander | 23 (16%) | 6 (18%) | 6 (13%) |  |
| Black | 3 (2.1%) | 3 (9.1%) | 4 (8.3%) |  |
| Hispanic | 34 (24%) | 9 (27%) | 12 (25%) |  |
| Other/Multiracial | 3 (2.1%) | 0 (0%) | 2 (4.2%) |  |
| White | 78 (55%) | 15 (45%) | 24 (50%) |  |
| Infant Sex |  |  |  | 0.2 |
| Female | 68 (48%) | 15 (45%) | 16 (33%) |  |
| Male | 73 (52%) | 18 (55%) | 32 (67%) |  |
| Gestational Age at Sample Collection (weeks) | 31.41 (3.1) | 31.39 (3.0) | 31.36 (3.3) | >0.9 |

*Acronyms: Early Autism Risk Longitudinal Investigation (EARLI), Markers of Autism Risk in Babies-Learning Early Signs (MARBLES)*

**Table S8.** Among participants with two urine metals measures (trimester 1/trimester 2 and trimester 3 pregnancy), cross timepoint Spearman correlations for each metal concentration, stratified by cohort (EARLI, MARBLES).

|  | **EARLI (n=137)** | **MARBLES (n=142)** |
| --- | --- | --- |
| Antimony | 0.33 | 0.26 |
| Arsenic | 0.42 | 0.47 |
| Barium | 0.42 | 0.21 |
| Cadmium | 0.47 | 0.31 |
| Cesium | 0.53 | 0.58 |
| Chromium | 0.36 | 0.4 |
| Cobalt | 0.19 | 0.3 |
| Copper | 0.36 | 0.31 |
| Lead | 0.43 | 0.19 |
| Manganese | 0.12 | -0.07 |
| Mercury | 0.5 | 0.43 |
| Molybdenum | 0.3 | 0.09 |
| Nickel | 0.24 | 0.18 |
| Selenium | 0.32 | 0.3 |
| Thallium | 0.41 | 0.38 |
| Tin | 0.59 | 0.53 |
| Zinc | 0.56 | 0.51 |

*Acronyms: Early Autism Risk Longitudinal Investigation (EARLI), Markers of Autism Risk in Babies-Learning Early Signs (MARBLES)*

**Table S9.** Adjusted risk ratios for the associations between urinary metal concentrations measured during pregnancy and risk of autism spectrum disorder, relative to typically developing. Log binomial models were adjusted for gestational age at sample collection, child sex, age at pregnancy, self-report race/ethnicity, and education. Four metals were modeled categorically (above versus below the limit of detection) and the remaining metals were log_2_ transformed and modeled continuously. *Symbols: ** meta-analysis p-value < 0.05. * meta-analysis p-value < 0.1.*

|  | | **EARLI** | | | **MARBLES** | | | **Meta-Analysis** | | | |
| --- | --- | --- | --- | --- | --- | --- | --- | --- | --- | --- | --- |
| **Metal** | **Time** | **RR** | **CI** | **P** | **RR** | **CI** | **P** | **RR** | **CI** | **P** | **FDR** |
| **Modeled categorically** | | | | | | | | | | | |
| Antimony | T1/T2 | 1.45 | (0.68,3.06) | 0.34 | 1.13 | (0.54,2.37) | 0.74 | 1.28 | (0.75,2.16) | 0.36 | 0.61 |
|  | T3 | 0.77 | (0.34,1.75) | 0.54 | 0.86 | (0.4,1.84) | 0.69 | 0.82 | (0.47,1.43) | 0.48 | 0.85 |
| Cadmium | T1/T2** | 1.85 | (0.9,3.81) | 0.095 | 1.6 | (0.91,2.82) | 0.1 | 1.69 | (1.08,2.64) | 0.021 | 0.18 |
|  | T3 | 1.39 | (0.76,2.55) | 0.28 | 0.93 | (0.52,1.67) | 0.82 | 1.13 | (0.75,1.72) | 0.56 | 0.91 |
| Chromium | T1/T2 | 1.06 | (0.5,2.26) | 0.88 | 1.33 | (0.68,2.58) | 0.41 | 1.2 | (0.73,1.98) | 0.47 | 0.71 |
|  | T3 | 1.3 | (0.64,2.64) | 0.47 | 0.94 | (0.42,2.09) | 0.88 | 1.13 | (0.66,1.91) | 0.66 | 0.94 |
| Lead | T1/T2 | 1.83 | (0.84,3.98) | 0.13 | 1.07 | (0.59,1.96) | 0.82 | 1.31 | (0.81,2.11) | 0.27 | 0.61 |
|  | T3 | 1.69 | (0.81,3.5) | 0.16 | 1.09 | (0.67,1.78) | 0.72 | 1.25 | (0.83,1.88) | 0.28 | 0.79 |
| Modeled continuously | | | | | | | | | | | |
| Arsenic | T1/T2** | 0.8 | (0.69,0.91) | 0.0012 | 1.0 | (0.77,1.29) | 0.99 | 0.84 | (0.74,0.94) | 0.0041 | 0.046 |
|  | T3 | 1.19 | (0.92,1.55) | 0.19 | 1.12 | (0.89,1.4) | 0.34 | 1.15 | (0.97,1.36) | 0.12 | 0.68 |
| Barium | T1/T2 | 1.32 | (0.94,1.86) | 0.11 | 1.08 | (0.85,1.39) | 0.52 | 1.16 | (0.95,1.42) | 0.15 | 0.46 |
|  | T3 | 0.98 | (0.76,1.25) | 0.86 | 1.0 | (0.84,1.2) | 0.97 | 0.99 | (0.86,1.15) | 0.94 | 0.98 |
| Cesium | T1/T2* | 4.38 | (0.97,19.82) | 0.055 | 1.5 | (0.68,3.3) | 0.32 | 1.89 | (0.94,3.8) | 0.075 | 0.39 |
|  | T3* | 1.77 | (0.7,4.46) | 0.23 | 1.65 | (0.83,3.31) | 0.16 | 1.69 | (0.97,2.95) | 0.063 | 0.43 |
| Cobalt | T1/T2 | 1.06 | (0.68,1.66) | 0.79 | 0.77 | (0.44,1.34) | 0.36 | 0.94 | (0.66,1.33) | 0.71 | 0.76 |
|  | T3 | 0.94 | (0.62,1.44) | 0.79 | 0.98 | (0.71,1.35) | 0.91 | 0.97 | (0.75,1.25) | 0.8 | 0.98 |
| Copper | T1/T2 | 2.03 | (0.64,6.38) | 0.23 | 1.21 | (0.65,2.24) | 0.55 | 1.36 | (0.79,2.34) | 0.27 | 0.61 |
|  | T3 | 3.15 | (0.98,10.15) | 0.055 | 0.94 | (0.71,1.25) | 0.67 | 1.01 | (0.76,1.33) | 0.97 | 0.98 |
| Manganese | T1/T2 | 0.9 | (0.56,1.43) | 0.65 | 1.04 | (0.78,1.39) | 0.77 | 1.0 | (0.78,1.28) | 0.99 | 0.99 |
|  | T3 | 0.98 | (0.73,1.33) | 0.91 | 1.04 | (0.83,1.32) | 0.72 | 1.02 | (0.85,1.23) | 0.83 | 0.98 |
| Mercury | T1/T2 | 1.12 | (0.93,1.35) | 0.23 | 0.83 | (0.6,1.14) | 0.25 | 1.04 | (0.88,1.22) | 0.65 | 0.76 |
|  | T3 | 1.14 | (1,1.3) | 0.048 | 0.92 | (0.73,1.16) | 0.47 | 1.08 | (0.97,1.21) | 0.18 | 0.76 |
| Molybdenum | T1/T2 | 1.92 | (0.81,4.55) | 0.14 | 0.98 | (0.67,1.42) | 0.9 | 1.09 | (0.77,1.53) | 0.63 | 0.76 |
|  | T3 | 0.92 | (0.78,1.09) | 0.33 | 1.07 | (0.77,1.48) | 0.68 | 0.95 | (0.82,1.1) | 0.49 | 0.85 |
| Nickel | T1/T2 | 1.3 | (0.74,2.28) | 0.37 | 1.13 | (0.57,2.25) | 0.72 | 1.23 | (0.79,1.9) | 0.36 | 0.61 |
|  | T3 | 0.99 | (0.64,1.52) | 0.96 | 0.99 | (0.69,1.43) | 0.97 | 0.99 | (0.75,1.31) | 0.95 | 0.98 |
| Selenium | T1/T2** | 1.46 | (0.37,5.75) | 0.59 | 0.88 | (0.83,0.94) | 0.00025 | 0.89 | (0.83,0.95) | 0.00028 | 0.0095 |
|  | T3 | 3.4 | (0.98,11.78) | 0.054 | 0.93 | (0.7,1.24) | 0.62 | 0.99 | (0.75,1.31) | 0.96 | 0.98 |
| Thallium | T1/T2** | 1.02 | (0.65,1.61) | 0.92 | 1.17 | (1.06,1.3) | 0.0023 | 1.16 | (1.05,1.28) | 0.0028 | 0.046 |
|  | T3** | 0.87 | (0.5,1.53) | 0.64 | 1.17 | (1.08,1.26) | 0.000072 | 1.16 | (1.08,1.25) | 0.00011 | 0.0037 |
| Tin | T1/T2 | 1.09 | (0.87,1.37) | 0.43 | 1.0 | (0.79,1.28) | 0.97 | 1.05 | (0.89,1.24) | 0.55 | 0.75 |
|  | T3** | 1.19 | (1.03,1.36) | 0.015 | 1.03 | (0.86,1.23) | 0.78 | 1.13 | (1.01,1.26) | 0.035 | 0.4 |
| Zinc | T1/T2 | 1.52 | (0.73,3.16) | 0.26 | 0.95 | (0.8,1.12) | 0.53 | 0.97 | (0.83,1.14) | 0.72 | 0.76 |
|  | T3 | 1.28 | (0.62,2.64) | 0.5 | 1.09 | (0.8,1.5) | 0.58 | 1.12 | (0.84,1.5) | 0.44 | 0.85 |

**Table S10.** Adjusted risk ratios for the associations between urinary metal concentrations measured during pregnancy and risk of non-typically developing, relative to typically developing. Log binomial models were adjusted for gestational age at sample collection, child sex, age at pregnacy, self-report race/ethnicity, and education. Four metals were modeled categorically (above versus below the limit of detection) and the remaining metals were log_2_ transformed and modeled continuously. *Symbols:* *** meta-analysis p-value < 0.05. * meta-analysis p-value < 0.1.*

|  | | **EARLI** | | | **MARBLES** | | | **Meta-Analysis** | | | |
| --- | --- | --- | --- | --- | --- | --- | --- | --- | --- | --- | --- |
| **Metal** | **Time** | **RR** | **CI** | **P** | **RR** | **CI** | **P** | **RR** | **CI** | **P** | **FDR** |
| **Modeled categorically** | | | | | | | | | | | |
| Antimony | T1/T2 | 0.95 | (0.64,1.42) | 0.81 | 0.37 | (0.05,2.63) | 0.32 | 0.92 | (0.62,1.36) | 0.66 | 0.76 |
|  | T3 | 0.96 | (0.66,1.41) | 0.85 | 1.66 | (0.82,3.37) | 0.16 | 1.09 | (0.78,1.53) | 0.62 | 0.92 |
| Cadmium | T1/T2 | 1.14 | (0.82,1.58) | 0.44 | 3.34 | (1.37,8.14) | 0.008 | 1.29 | (0.95,1.75) | 0.1 | 0.41 |
|  | T3 | 1.03 | (0.75,1.42) | 0.86 | 2.01 | (1.08,3.77) | 0.028 | 1.18 | (0.89,1.57) | 0.25 | 0.79 |
| Chromium | T1/T2 | 1.04 | (0.73,1.48) | 0.83 | 1.5 | (0.54,4.16) | 0.44 | 1.08 | (0.78,1.5) | 0.65 | 0.76 |
|  | T3 | 1.05 | (0.7,1.57) | 0.82 | 1.49 | (0.69,3.22) | 0.31 | 1.13 | (0.79,1.61) | 0.5 | 0.85 |
| Lead | T1/T2 | 1.05 | (0.75,1.47) | 0.79 | 2.38 | (0.96,5.89) | 0.062 | 1.16 | (0.84,1.59) | 0.36 | 0.61 |
|  | T3 | 0.82 | (0.59,1.13) | 0.22 | 1.1 | (0.59,2.06) | 0.76 | 0.87 | (0.65,1.16) | 0.34 | 0.85 |
| **Modeled continuously** | | | | | | | | | | | |
| Arsenic | T1/T2 | 1.09 | (0.93,1.27) | 0.28 | 0.86 | (0.59,1.25) | 0.42 | 1.05 | (0.91,1.21) | 0.48 | 0.71 |
|  | T3 | 1.08 | (0.92,1.26) | 0.36 | 0.8 | (0.67,0.95) | 0.0098 | 0.94 | (0.83,1.05) | 0.28 | 0.79 |
| Barium | T1/T2 | 1.01 | (0.88,1.15) | 0.92 | 1.08 | (0.72,1.61) | 0.72 | 1.01 | (0.89,1.15) | 0.84 | 0.87 |
|  | T3 | 0.93 | (0.84,1.04) | 0.23 | 0.97 | (0.76,1.23) | 0.77 | 0.94 | (0.85,1.04) | 0.23 | 0.79 |
| Cesium | T1/T2 | 1.51 | (0.84,2.74) | 0.17 | 1.44 | (0.53,3.92) | 0.48 | 1.49 | (0.9,2.49) | 0.12 | 0.41 |
|  | T3 | 0.98 | (0.79,1.22) | 0.87 | 1.17 | (0.58,2.39) | 0.66 | 1.0 | (0.81,1.23) | 0.98 | 0.98 |
| Cobalt | T1/T2 | 1.09 | (0.91,1.3) | 0.37 | 1.12 | (0.54,2.32) | 0.77 | 1.09 | (0.91,1.3) | 0.35 | 0.61 |
|  | T3 | 0.86 | (0.68,1.09) | 0.21 | 0.84 | (0.54,1.29) | 0.42 | 0.85 | (0.69,1.05) | 0.14 | 0.68 |
| Copper | T1/T2 | 1.05 | (0.73,1.53) | 0.78 | 1.68 | (0.63,4.51) | 0.3 | 1.12 | (0.79,1.59) | 0.53 | 0.75 |
|  | T3 | 0.98 | (0.78,1.22) | 0.85 | 0.87 | (0.64,1.18) | 0.37 | 0.94 | (0.78,1.12) | 0.5 | 0.85 |
| Manganese | T1/T2 | 0.87 | (0.66,1.13) | 0.3 | 0.98 | (0.61,1.58) | 0.94 | 0.89 | (0.71,1.13) | 0.34 | 0.61 |
|  | T3 | 0.92 | (0.77,1.11) | 0.4 | 1.06 | (0.8,1.4) | 0.7 | 0.96 | (0.82,1.12) | 0.62 | 0.92 |
| Mercury | T1/T2* | 1.07 | (1,1.15) | 0.05 | 0.73 | (0.45,1.18) | 0.19 | 1.06 | (0.99,1.14) | 0.08 | 0.39 |
|  | T3 | 1.05 | (0.97,1.14) | 0.22 | 0.78 | (0.57,1.08) | 0.14 | 1.03 | (0.96,1.12) | 0.4 | 0.85 |
| Molybdenum | T1/T2 | 1.46 | (0.86,2.48) | 0.16 | 1.32 | (0.59,2.96) | 0.51 | 1.42 | (0.91,2.21) | 0.12 | 0.41 |
|  | T3 | 1.01 | (0.83,1.23) | 0.92 | 0.95 | (0.71,1.27) | 0.72 | 0.99 | (0.84,1.17) | 0.91 | 0.98 |
| Nickel | T1/T2* | 1.56 | (1.06,2.3) | 0.025 | 0.91 | (0.43,1.93) | 0.81 | 1.39 | (0.99,1.96) | 0.061 | 0.39 |
|  | T3 | 1.14 | (0.86,1.52) | 0.35 | 0.76 | (0.58,1) | 0.046 | 0.92 | (0.76,1.12) | 0.42 | 0.85 |
| Selenium | T1/T2 | 1.38 | (0.75,2.56) | 0.3 | 1.14 | (0.53,2.47) | 0.73 | 1.28 | (0.79,2.08) | 0.31 | 0.61 |
|  | T3** | 1.2 | (0.61,2.36) | 0.6 | 0.86 | (0.75,0.99) | 0.034 | 0.87 | (0.76,1.0) | 0.049 | 0.42 |
| Thallium | T1/T2 | 0.62 | (0.32,1.19) | 0.15 | 0.93 | (0.52,1.68) | 0.82 | 0.78 | (0.5,1.2) | 0.26 | 0.61 |
|  | T3 | 0.89 | (0.63,1.24) | 0.48 | 1.04 | (0.73,1.49) | 0.82 | 0.96 | (0.75,1.22) | 0.72 | 0.98 |
| Tin | T1/T2 | 1.05 | (0.95,1.16) | 0.3 | 0.78 | (0.48,1.26) | 0.31 | 1.04 | (0.94,1.15) | 0.42 | 0.68 |
|  | T3 | 1.03 | (0.93,1.13) | 0.6 | 0.79 | (0.57,1.09) | 0.15 | 1.0 | (0.91,1.1) | 0.93 | 0.98 |
| Zinc | T1/T2 | 0.98 | (0.87,1.11) | 0.77 | 0.94 | (0.66,1.34) | 0.75 | 0.98 | (0.88,1.09) | 0.71 | 0.76 |
|  | T3** | 0.97 | (0.94,1.0) | 0.026 | 0.92 | (0.81,1.04) | 0.18 | 0.97 | (0.94,0.99) | 0.014 | 0.24 |

**Table S11.** Pregnant person and child characteristics of participants in the sensitivity analytic sample with measures of metal exposure in blood from pregnancy in the Early Autism Risk Longitudinal Investigation (EARLI) cohort. Data are comparted by neurodevelopmental status of the sibling. Distributions of categorical variables are compared with a chi-square test and continuous variables are compared with an ANOVA test.

|  | **Typically developing** | **Non-typically developing** | **Autism spectrum disorder** | **P-value** |
| --- | --- | --- | --- | --- |
|  | ***N=41*** | ***N=32*** | ***N=19*** |  |
| Education |  |  |  | 0.175 |
| College Degree | 28 (68.3%) | 16 (50.0%) | 9 (47.4%) |  |
| No Degree | 13 (31.7%) | 16 (50.0%) | 10 (52.6%) |  |
| Age (years) | 34.4 ± 4.99 | 33.0 ± 4.73 | 33.5 ± 3.95 | 0.446 |
| Infant Sex |  |  |  | 0.001 |
| Female | 24 (58.5%) | 20 (62.5%) | 3 (15.8%) |  |
| Male | 17 (41.5%) | 12 (37.5%) | 16 (84.2%) |  |
| Infant Gestational Age at Birth (weeks) | 39.5 ± 1.51 | 39.3 ± 1.69 | 39.1 ± 1.68 | 0.651 |

**Table S12.** Batch adjusted risk ratios for the associations between urinary metal concentrations measured during pregnancy and risk of autism spectrum disorder, relative to typically developing. Log binomial models were adjusted for batch, gestational age at sample collection, child sex, age at pregnancy, self-report race/ethnicity, and education. Four metals were modeled categorically (above versus below the limit of detection) and the remaining metals were log_2_ transformed and modeled continuously.

|  | | **EARLI** | | | **MARBLES** | | | **Meta-Analysis** | | | |
| --- | --- | --- | --- | --- | --- | --- | --- | --- | --- | --- | --- |
| **Metal** | **Time** | **RR** | **CI** | **P** | **RR** | **CI** | **P** | **RR** | **CI** | **P** | **FDR** |
| **Modeled categorically** | | | | | | | | | | | |
| Antimony | T1/T2* | 2.03 | (0.96,4.29) | 0.063 | 1.35 | (0.67,2.73) | 0.4 | 1.64 | (0.98,2.73) | 0.059 | 0.39 |
|  | T3 | 0.85 | (0.37,1.95) | 0.7 | 0.94 | (0.44,1.98) | 0.86 | 0.9 | (0.51,1.57) | 0.7 | 0.92 |
| Cadmium | T1/T2** | 1.83 | (0.89,3.76) | 0.099 | 1.6 | (0.91,2.8) | 0.1 | 1.68 | (1.08,2.62) | 0.021 | 0.18 |
|  | T3 | 1.43 | (0.78,2.6) | 0.25 | 0.87 | (0.48,1.56) | 0.64 | 1.1 | (0.73,1.68) | 0.64 | 0.92 |
| Chromium | T1/T2 | 0.94 | (0.43,2.04) | 0.87 | 1.17 | (0.59,2.32) | 0.65 | 1.06 | (0.64,1.78) | 0.81 | 0.82 |
|  | T3 | 1.28 | (0.63,2.6) | 0.5 | 0.92 | (0.42,2.04) | 0.84 | 1.11 | (0.65,1.88) | 0.71 | 0.92 |
| Lead | T1/T2 | 1.72 | (0.8,3.7) | 0.17 | 1.03 | (0.57,1.87) | 0.93 | 1.25 | (0.78,2) | 0.35 | 0.61 |
|  | T3 | 1.62 | (0.78,3.4) | 0.2 | 1.04 | (0.64,1.7) | 0.87 | 1.19 | (0.79,1.79) | 0.4 | 0.92 |
| Modeled continuously | | | | | | | | | | | |
| Arsenic | T1/T2** | 0.8 | (0.71,0.89) | 0.000086 | 0.96 | (0.76,1.21) | 0.72 | 0.82 | (0.75,0.91) | 0.00021 | 0.0036 |
|  | T3 | 1.2 | (0.92,1.57) | 0.18 | 1.09 | (0.88,1.36) | 0.43 | 1.13 | (0.96,1.34) | 0.15 | 0.85 |
| Barium | T1/T2 | 1.26 | (0.89,1.78) | 0.19 | 1.09 | (0.85,1.39) | 0.51 | 1.14 | (0.93,1.39) | 0.19 | 0.54 |
|  | T3 | 0.92 | (0.72,1.18) | 0.53 | 1 | (0.84,1.2) | 0.99 | 0.97 | (0.84,1.13) | 0.72 | 0.92 |
| Cesium | T1/T2 | 3.6 | (0.78,16.54) | 0.1 | 1.45 | (0.67,3.17) | 0.35 | 1.75 | (0.88,3.51) | 0.11 | 0.4 |
|  | T3* | 1.65 | (0.65,4.19) | 0.29 | 1.59 | (0.8,3.16) | 0.18 | 1.61 | (0.93,2.8) | 0.089 | 0.76 |
| Cobalt | T1/T2 | 1.1 | (0.72,1.68) | 0.66 | 0.76 | (0.44,1.3) | 0.32 | 0.96 | (0.68,1.33) | 0.79 | 0.82 |
|  | T3 | 0.89 | (0.57,1.39) | 0.61 | 0.99 | (0.72,1.36) | 0.95 | 0.95 | (0.74,1.24) | 0.73 | 0.92 |
| Copper | T1/T2 | 1.5 | (0.49,4.6) | 0.48 | 1.3 | (0.67,2.53) | 0.44 | 1.35 | (0.76,2.39) | 0.3 | 0.61 |
|  | T3 | 2.99 | (0.89,9.99) | 0.076 | 0.95 | (0.71,1.26) | 0.72 | 1.01 | (0.76,1.33) | 0.95 | 0.95 |
| Manganese | T1/T2 | 0.69 | (0.35,1.35) | 0.28 | 1.0 | (0.74,1.36) | 1.0 | 0.94 | (0.71,1.24) | 0.66 | 0.77 |
|  | T3 | 0.87 | (0.57,1.34) | 0.54 | 1.05 | (0.84,1.32) | 0.65 | 1.01 | (0.83,1.24) | 0.9 | 0.95 |
| Mercury | T1/T2 | 1.05 | (0.8,1.37) | 0.74 | 0.79 | (0.57,1.1) | 0.16 | 0.94 | (0.76,1.15) | 0.54 | 0.76 |
|  | T3 | 1.13 | (0.95,1.33) | 0.16 | 0.9 | (0.71,1.14) | 0.37 | 1.04 | (0.91,1.2) | 0.53 | 0.92 |
| Molybdenum | T1/T2 | 2.44 | (0.94,6.35) | 0.067 | 1.03 | (0.65,1.62) | 0.91 | 1.21 | (0.8,1.82) | 0.37 | 0.61 |
|  | T3 | 0.93 | (0.76,1.15) | 0.52 | 1.06 | (0.78,1.45) | 0.71 | 0.97 | (0.82,1.16) | 0.75 | 0.92 |
| Nickel | T1/T2 | 1.52 | (0.79,2.96) | 0.21 | 1.15 | (0.57,2.33) | 0.7 | 1.34 | (0.82,2.17) | 0.24 | 0.61 |
|  | T3 | 1.0 | (0.65,1.55) | 0.99 | 1.02 | (0.7,1.47) | 0.93 | 1.01 | (0.76,1.34) | 0.94 | 0.95 |
| Selenium | T1/T2** | 1.91 | (0.4,9.17) | 0.42 | 0.89 | (0.83,0.94) | 0.000095 | 0.89 | (0.83,0.94) | 0.00011 | 0.0036 |
|  | T3 | 5.91 | (1.35,25.76) | 0.018 | 0.91 | (0.74,1.12) | 0.36 | 0.94 | (0.77,1.16) | 0.57 | 0.92 |
| Thallium | T1/T2 | 0.95 | (0.55,1.65) | 0.86 | 1.1 | (0.85,1.43) | 0.48 | 1.07 | (0.85,1.36) | 0.57 | 0.76 |
|  | T3 | 0.77 | (0.37,1.57) | 0.47 | 1.13 | (0.92,1.39) | 0.26 | 1.09 | (0.9,1.33) | 0.38 | 0.92 |
| Tin | T1/T2 | 1.08 | (0.85,1.36) | 0.54 | 0.98 | (0.77,1.26) | 0.89 | 1.03 | (0.87,1.22) | 0.73 | 0.8 |
|  | T3* | 1.18 | (1.02,1.36) | 0.029 | 1.01 | (0.84,1.22) | 0.92 | 1.11 | (0.99,1.25) | 0.073 | 0.76 |
| Zinc | T1/T2 | 1.36 | (0.65,2.85) | 0.41 | 0.95 | (0.82,1.1) | 0.48 | 0.96 | (0.83,1.11) | 0.6 | 0.76 |
|  | T3 | 1.19 | (0.59,2.42) | 0.62 | 1.07 | (0.79,1.46) | 0.65 | 1.09 | (0.82,1.44) | 0.54 | 0.92 |

*Symbols: ** meta-analysis p-value < 0.05. * meta-analysis p-value < 0.1.*

**Table S13.** Batch adjusted risk ratios for the associations between urinary metal concentrations measured during pregnancy and risk of non-typically developing, relative to typically developing. Log binomial models were adjusted for batch, gestational age at sample collection, child sex, age at pregnancy, self-report race/ethnicity, and education. Four metals were modeled categorically (above versus below the limit of detection) and the remaining metals were log_2_ transformed and modeled continuously.

|  | | **EARLI** | | | **MARBLES** | | | **Meta-Analysis** | | | |
| --- | --- | --- | --- | --- | --- | --- | --- | --- | --- | --- | --- |
| **Metal** | **Time** | **RR** | **CI** | **P** | **RR** | **CI** | **P** | **RR** | **CI** | **P** | **FDR** |
| **Modeled categorically** | | | | | | | | | | | |
| Antimony | T1/T2 | 0.81 | (0.49,1.34) | 0.42 | 0.47 | (0.07,3.25) | 0.45 | 0.78 | (0.48,1.28) | 0.33 | 0.61 |
|  | T3 | 0.86 | (0.55,1.36) | 0.53 | 1.71 | (0.85,3.47) | 0.13 | 1.05 | (0.72,1.54) | 0.79 | 0.93 |
| Cadmium | T1/T2 | 1.12 | (0.8,1.55) | 0.51 | 3.27 | (1.34,8) | 0.0094 | 1.27 | (0.93,1.73) | 0.13 | 0.4 |
|  | T3 | 1.01 | (0.73,1.4) | 0.93 | 2.13 | (1.14,3.98) | 0.018 | 1.19 | (0.89,1.58) | 0.24 | 0.92 |
| Chromium | T1/T2 | 1.06 | (0.75,1.5) | 0.75 | 1.34 | (0.47,3.79) | 0.58 | 1.09 | (0.78,1.51) | 0.63 | 0.76 |
|  | T3 | 1.05 | (0.7,1.56) | 0.83 | 1.59 | (0.74,3.39) | 0.23 | 1.15 | (0.8,1.63) | 0.45 | 0.92 |
| Lead | T1/T2 | 1.05 | (0.75,1.48) | 0.77 | 2.27 | (0.92,5.61) | 0.076 | 1.16 | (0.84,1.59) | 0.37 | 0.61 |
|  | T3 | 0.83 | (0.6,1.14) | 0.25 | 1.09 | (0.58,2.04) | 0.8 | 0.88 | (0.66,1.17) | 0.37 | 0.92 |
| Modeled continuously | | | | | | | | | | | |
| Arsenic | T1/T2 | 1.09 | (0.93,1.27) | 0.29 | 0.82 | (0.58,1.17) | 0.28 | 1.04 | (0.9,1.2) | 0.58 | 0.76 |
|  | T3 | 1.07 | (0.92,1.26) | 0.39 | 0.8 | (0.67,0.95) | 0.012 | 0.94 | (0.84,1.06) | 0.3 | 0.92 |
| Barium | T1/T2 | 1.02 | (0.89,1.16) | 0.81 | 1.08 | (0.73,1.61) | 0.69 | 1.02 | (0.9,1.16) | 0.72 | 0.8 |
|  | T3 | 0.94 | (0.84,1.07) | 0.35 | 0.97 | (0.76,1.23) | 0.77 | 0.95 | (0.85,1.06) | 0.33 | 0.92 |
| Cesium | T1/T2 | 1.53 | (0.84,2.76) | 0.16 | 1.4 | (0.52,3.76) | 0.5 | 1.49 | (0.9,2.48) | 0.12 | 0.4 |
|  | T3 | 1.01 | (0.79,1.29) | 0.94 | 1.16 | (0.57,2.36) | 0.68 | 1.02 | (0.81,1.29) | 0.84 | 0.94 |
| Cobalt | T1/T2 | 1.08 | (0.89,1.3) | 0.42 | 1.09 | (0.53,2.22) | 0.82 | 1.08 | (0.9,1.3) | 0.41 | 0.61 |
|  | T3 | 0.87 | (0.69,1.11) | 0.28 | 0.84 | (0.55,1.29) | 0.43 | 0.87 | (0.7,1.07) | 0.18 | 0.87 |
| Copper | T1/T2 | 1.15 | (0.68,1.94) | 0.6 | 1.9 | (0.68,5.3) | 0.22 | 1.28 | (0.8,2.03) | 0.3 | 0.61 |
|  | T3 | 1.05 | (0.72,1.54) | 0.79 | 0.87 | (0.64,1.19) | 0.39 | 0.94 | (0.74,1.2) | 0.62 | 0.92 |
| Manganese | T1/T2 | 0.88 | (0.66,1.18) | 0.39 | 0.94 | (0.58,1.53) | 0.8 | 0.89 | (0.7,1.15) | 0.38 | 0.61 |
|  | T3 | 0.94 | (0.75,1.18) | 0.6 | 1.06 | (0.8,1.4) | 0.7 | 0.98 | (0.83,1.17) | 0.86 | 0.94 |
| Mercury | T1/T2** | 1.08 | (1.02,1.15) | 0.0083 | 0.7 | (0.43,1.13) | 0.14 | 1.08 | (1.01,1.14) | 0.015 | 0.17 |
|  | T3** | 1.08 | (1.02,1.13) | 0.0039 | 0.76 | (0.54,1.07) | 0.12 | 1.07 | (1.02,1.12) | 0.0086 | 0.15 |
| Molybdenum | T1/T2 | 1.45 | (0.83,2.54) | 0.19 | 1.39 | (0.61,3.17) | 0.43 | 1.43 | (0.9,2.28) | 0.13 | 0.4 |
|  | T3 | 1 | (0.83,1.19) | 0.97 | 0.93 | (0.71,1.22) | 0.62 | 0.98 | (0.84,1.13) | 0.76 | 0.92 |
| Nickel | T1/T2* | 1.57 | (1.04,2.37) | 0.031 | 0.94 | (0.43,2.03) | 0.87 | 1.4 | (0.97,2.01) | 0.068 | 0.39 |
|  | T3 | 1.13 | (0.86,1.49) | 0.39 | 0.77 | (0.58,1.01) | 0.055 | 0.93 | (0.76,1.13) | 0.45 | 0.92 |
| Selenium | T1/T2 | 1.33 | (0.69,2.57) | 0.39 | 1.11 | (0.51,2.39) | 0.79 | 1.23 | (0.75,2.03) | 0.41 | 0.61 |
|  | T3 | 1.16 | (0.6,2.24) | 0.66 | 0.86 | (0.72,1.03) | 0.098 | 0.88 | (0.74,1.04) | 0.14 | 0.85 |
| Thallium | T1/T2 | 0.64 | (0.33,1.24) | 0.19 | 0.73 | (0.35,1.53) | 0.41 | 0.68 | (0.41,1.11) | 0.13 | 0.4 |
|  | T3 | 0.92 | (0.66,1.28) | 0.62 | 0.99 | (0.61,1.62) | 0.98 | 0.94 | (0.72,1.24) | 0.67 | 0.92 |
| Tin | T1/T2 | 1.06 | (0.96,1.17) | 0.25 | 0.73 | (0.43,1.22) | 0.23 | 1.04 | (0.95,1.15) | 0.37 | 0.61 |
|  | T3 | 1.04 | (0.94,1.14) | 0.44 | 0.77 | (0.55,1.08) | 0.13 | 1.02 | (0.93,1.11) | 0.74 | 0.92 |
| Zinc | T1/T2 | 0.99 | (0.86,1.15) | 0.9 | 0.95 | (0.67,1.34) | 0.76 | 0.98 | (0.86,1.13) | 0.82 | 0.82 |
|  | T3** | 0.97 | (0.94,0.99) | 0.015 | 0.92 | (0.81,1.04) | 0.16 | 0.97 | (0.94,0.99) | 0.0075 | 0.15 |

*Symbols: ** meta-analysis p-value < 0.05. * meta-analysis p-value < 0.1.*

**Table S14.** Adjusted odds ratios for the associations between urinary metal concentrations measured during pregnancy and odds of autism spectrum disorder, relative to typically developing. Logistic regression models were adjusted for gestational age at sample collection, child sex, age at pregnancy, self-report race/ethnicity, and education. Four metals were modeled categorically (above versus below the limit of detection) and the remaining metals were log_2_ transformed and modeled continuously.

|  | | **EARLI** | | | **MARBLES** | | | **Meta-Analysis** | | | |
| --- | --- | --- | --- | --- | --- | --- | --- | --- | --- | --- | --- |
| **Metal** | **Time** | **OR** | **CI** | **P** | **OR** | **CI** | **P** | **OR** | **CI** | **P** | **FDR** |
| **Modeled categorically** | | | | | | | | | | | |
| Antimony | T1/T2 | 1.95 | (0.49,7.71) | 0.34 | 1.2 | (0.41,3.52) | 0.74 | 1.44 | (0.62,3.37) | 0.4 | 0.68 |
|  | T3 | 0.64 | (0.16,2.53) | 0.52 | 0.81 | (0.29,2.26) | 0.69 | 0.74 | (0.33,1.7) | 0.48 | 0.96 |
| Cadmium | T1/T2** | 3.01 | (0.84,10.75) | 0.09 | 1.99 | (0.85,4.67) | 0.11 | 2.26 | (1.12,4.59) | 0.024 | 0.3 |
|  | T3 | 1.83 | (0.6,5.57) | 0.29 | 0.91 | (0.41,2.02) | 0.82 | 1.16 | (0.6,2.21) | 0.66 | 0.96 |
| Chromium | T1/T2 | 1.11 | (0.28,4.4) | 0.88 | 1.51 | (0.55,4.13) | 0.42 | 1.36 | (0.6,3.06) | 0.46 | 0.71 |
|  | T3 | 1.63 | (0.4,6.68) | 0.49 | 0.92 | (0.31,2.74) | 0.88 | 1.14 | (0.48,2.71) | 0.76 | 0.96 |
| Lead | T1/T2 | 2.97 | (0.73,11.98) | 0.13 | 1.11 | (0.47,2.61) | 0.82 | 1.45 | (0.7,3.01) | 0.32 | 0.66 |
|  | T3 | 2.49 | (0.73,8.48) | 0.14 | 1.13 | (0.57,2.25) | 0.72 | 1.37 | (0.75,2.49) | 0.31 | 0.96 |
| Modeled continuously | | | | | | | | | | | |
| Arsenic | T1/T2 | 0.54 | (0.3,0.99) | 0.048 | 1.0 | (0.69,1.44) | 0.99 | 0.85 | (0.62,1.16) | 0.3 | 0.66 |
|  | T3* | 1.31 | (0.92,1.87) | 0.14 | 1.15 | (0.88,1.5) | 0.3 | 1.21 | (0.97,1.49) | 0.086 | 0.82 |
| Barium | T1/T2 | 1.63 | (0.9,2.97) | 0.11 | 1.12 | (0.79,1.58) | 0.52 | 1.23 | (0.91,1.66) | 0.17 | 0.62 |
|  | T3 | 0.96 | (0.61,1.52) | 0.86 | 1.0 | (0.78,1.29) | 0.97 | 0.99 | (0.8,1.24) | 0.96 | 0.96 |
| Cesium | T1/T2** | 5.82 | (1.38,24.59) | 0.017 | 1.62 | (0.7,3.74) | 0.26 | 2.24 | (1.09,4.61) | 0.029 | 0.3 |
|  | T3** | 2.24 | (0.76,6.61) | 0.15 | 1.79 | (0.88,3.67) | 0.11 | 1.92 | (1.06,3.49) | 0.033 | 0.82 |
| Cobalt | T1/T2 | 1.12 | (0.47,2.68) | 0.8 | 0.7 | (0.34,1.43) | 0.33 | 0.85 | (0.49,1.47) | 0.55 | 0.76 |
|  | T3 | 0.9 | (0.43,1.89) | 0.79 | 0.97 | (0.62,1.53) | 0.9 | 0.95 | (0.65,1.4) | 0.81 | 0.96 |
| Copper | T1/T2 | 2.38 | (0.77,7.4) | 0.13 | 1.26 | (0.64,2.5) | 0.51 | 1.49 | (0.83,2.68) | 0.18 | 0.62 |
|  | T3 | 3.34 | (1.14,9.77) | 0.028 | 0.91 | (0.57,1.45) | 0.7 | 1.12 | (0.73,1.71) | 0.61 | 0.96 |
| Manganese | T1/T2 | 0.84 | (0.43,1.66) | 0.62 | 1.06 | (0.69,1.65) | 0.78 | 0.99 | (0.69,1.44) | 0.97 | 0.97 |
|  | T3 | 0.97 | (0.58,1.63) | 0.91 | 1.06 | (0.75,1.51) | 0.73 | 1.03 | (0.77,1.38) | 0.82 | 0.96 |
| Mercury | T1/T2 | 1.28 | (0.77,2.14) | 0.34 | 0.79 | (0.55,1.13) | 0.2 | 0.93 | (0.69,1.25) | 0.62 | 0.78 |
|  | T3 | 1.4 | (0.82,2.38) | 0.22 | 0.9 | (0.68,1.19) | 0.45 | 0.99 | (0.77,1.26) | 0.92 | 0.96 |
| Molybdenum | T1/T2 | 2.01 | (0.93,4.35) | 0.075 | 0.97 | (0.54,1.74) | 0.91 | 1.27 | (0.79,2.02) | 0.32 | 0.66 |
|  | T3 | 0.82 | (0.44,1.53) | 0.53 | 1.09 | (0.75,1.58) | 0.66 | 1.01 | (0.73,1.39) | 0.95 | 0.96 |
| Nickel | T1/T2 | 1.51 | (0.67,3.43) | 0.32 | 1.18 | (0.51,2.71) | 0.7 | 1.34 | (0.75,2.4) | 0.33 | 0.66 |
|  | T3 | 0.98 | (0.45,2.14) | 0.96 | 0.99 | (0.59,1.66) | 0.97 | 0.99 | (0.64,1.52) | 0.95 | 0.96 |
| Selenium | T1/T2 | 1.57 | (0.43,5.73) | 0.49 | 0.73 | (0.38,1.43) | 0.36 | 0.86 | (0.48,1.56) | 0.62 | 0.78 |
|  | T3 | 3.43 | (1.03,11.44) | 0.045 | 0.89 | (0.49,1.6) | 0.69 | 1.15 | (0.68,1.96) | 0.6 | 0.96 |
| Thallium | T1/T2 | 1.05 | (0.43,2.53) | 0.92 | 1.43 | (0.83,2.45) | 0.2 | 1.31 | (0.83,2.08) | 0.25 | 0.66 |
|  | T3 | 0.82 | (0.39,1.7) | 0.59 | 1.42 | (0.9,2.26) | 0.13 | 1.22 | (0.82,1.8) | 0.32 | 0.96 |
| Tin | T1/T2 | 1.2 | (0.73,1.98) | 0.48 | 1.01 | (0.72,1.42) | 0.97 | 1.06 | (0.8,1.41) | 0.67 | 0.81 |
|  | T3 | 1.49 | (0.97,2.27) | 0.066 | 1.04 | (0.8,1.35) | 0.78 | 1.15 | (0.92,1.44) | 0.23 | 0.96 |
| Zinc | T1/T2 | 1.58 | (0.82,3.04) | 0.17 | 0.91 | (0.61,1.35) | 0.64 | 1.05 | (0.75,1.48) | 0.76 | 0.83 |
|  | T3 | 1.33 | (0.69,2.59) | 0.4 | 1.11 | (0.79,1.55) | 0.54 | 1.15 | (0.85,1.55) | 0.35 | 0.96 |

*Symbols: ** meta-analysis p-value < 0.05. * meta-analysis p-value < 0.1.*

**Table S15.** Adjusted odds ratios for the associations between urinary metal concentrations measured during pregnancy and odds of non-typically developing, relative to typically developing. Logistic regression models were adjusted for gestational age at sample collection, child sex, age at pregnancy, self-report race/ethnicity, and education. Four metals were modeled categorically (above versus below the limit of detection) and the remaining metals were log_2_ transformed and modeled continuously.

|  | | **EARLI** | | | **MARBLES** | | | **Meta-Analysis** | | | |
| --- | --- | --- | --- | --- | --- | --- | --- | --- | --- | --- | --- |
| **Metal** | **Time** | **OR** | **CI** | **P** | **OR** | **CI** | **P** | **OR** | **CI** | **P** | **FDR** |
| **Modeled categorically** | | | | | | | | | | | |
| Antimony | T1/T2 | 0.89 | (0.36,2.21) | 0.81 | 0.33 | (0.04,2.71) | 0.3 | 0.76 | (0.33,1.76) | 0.52 | 0.76 |
|  | T3 | 0.92 | (0.38,2.21) | 0.85 | 1.94 | (0.74,5.08) | 0.18 | 1.29 | (0.67,2.46) | 0.45 | 0.96 |
| Cadmium | T1/T2** | 1.34 | (0.63,2.85) | 0.44 | 4.37 | (1.45,13.19) | 0.0089 | 1.95 | (1.05,3.63) | 0.035 | 0.3 |
|  | T3 | 1.07 | (0.51,2.25) | 0.86 | 2.48 | (1.07,5.73) | 0.034 | 1.55 | (0.89,2.71) | 0.12 | 0.82 |
| Chromium | T1/T2 | 1.1 | (0.48,2.49) | 0.83 | 1.63 | (0.46,5.84) | 0.45 | 1.23 | (0.62,2.45) | 0.56 | 0.76 |
|  | T3 | 1.12 | (0.43,2.94) | 0.82 | 1.68 | (0.59,4.77) | 0.33 | 1.35 | (0.67,2.74) | 0.4 | 0.96 |
| Lead | T1/T2 | 1.11 | (0.52,2.41) | 0.78 | 2.84 | (0.94,8.57) | 0.064 | 1.51 | (0.8,2.85) | 0.2 | 0.62 |
|  | T3 | 0.62 | (0.28,1.35) | 0.23 | 1.13 | (0.52,2.48) | 0.76 | 0.83 | (0.48,1.45) | 0.52 | 0.96 |
| Modeled continuously | | | | | | | | | | | |
| Arsenic | T1/T2 | 1.18 | (0.91,1.52) | 0.21 | 0.82 | (0.48,1.4) | 0.46 | 1.1 | (0.88,1.39) | 0.41 | 0.68 |
|  | T3 | 1.16 | (0.87,1.55) | 0.31 | 0.7 | (0.49,1.01) | 0.055 | 0.96 | (0.76,1.2) | 0.7 | 0.96 |
| Barium | T1/T2 | 1.01 | (0.75,1.38) | 0.92 | 1.09 | (0.68,1.75) | 0.72 | 1.04 | (0.8,1.34) | 0.78 | 0.83 |
|  | T3 | 0.84 | (0.63,1.13) | 0.25 | 0.96 | (0.71,1.3) | 0.78 | 0.9 | (0.73,1.11) | 0.31 | 0.96 |
| Cesium | T1/T2* | 1.98 | (0.92,4.29) | 0.081 | 1.49 | (0.53,4.21) | 0.45 | 1.79 | (0.97,3.33) | 0.065 | 0.37 |
|  | T3 | 0.96 | (0.56,1.63) | 0.88 | 1.21 | (0.54,2.7) | 0.65 | 1.03 | (0.66,1.6) | 0.9 | 0.96 |
| Cobalt | T1/T2 | 1.25 | (0.71,2.18) | 0.44 | 1.14 | (0.47,2.79) | 0.77 | 1.22 | (0.76,1.95) | 0.42 | 0.68 |
|  | T3 | 0.72 | (0.45,1.16) | 0.18 | 0.8 | (0.47,1.37) | 0.41 | 0.76 | (0.53,1.08) | 0.12 | 0.82 |
| Copper | T1/T2 | 1.12 | (0.56,2.23) | 0.76 | 1.72 | (0.65,4.54) | 0.27 | 1.29 | (0.73,2.27) | 0.38 | 0.68 |
|  | T3 | 0.95 | (0.52,1.73) | 0.86 | 0.81 | (0.47,1.41) | 0.46 | 0.87 | (0.58,1.31) | 0.51 | 0.96 |
| Manganese | T1/T2 | 0.78 | (0.53,1.14) | 0.19 | 0.98 | (0.56,1.7) | 0.94 | 0.84 | (0.61,1.15) | 0.27 | 0.66 |
|  | T3 | 0.85 | (0.62,1.17) | 0.33 | 1.07 | (0.73,1.57) | 0.71 | 0.94 | (0.73,1.2) | 0.61 | 0.96 |
| Mercury | T1/T2 | 1.24 | (0.91,1.69) | 0.17 | 0.71 | (0.44,1.15) | 0.17 | 1.06 | (0.81,1.37) | 0.69 | 0.81 |
|  | T3 | 1.16 | (0.85,1.58) | 0.35 | 0.76 | (0.54,1.07) | 0.11 | 0.95 | (0.76,1.2) | 0.69 | 0.96 |
| Molybdenum | T1/T2* | 1.58 | (0.97,2.55) | 0.064 | 1.33 | (0.6,2.95) | 0.48 | 1.51 | (1,2.28) | 0.051 | 0.35 |
|  | T3 | 1.02 | (0.67,1.56) | 0.91 | 0.93 | (0.6,1.45) | 0.75 | 0.98 | (0.72,1.33) | 0.88 | 0.96 |
| Nickel | T1/T2** | 2.15 | (1.24,3.72) | 0.0062 | 0.89 | (0.34,2.33) | 0.82 | 1.73 | (1.08,2.79) | 0.024 | 0.3 |
|  | T3 | 1.31 | (0.79,2.17) | 0.29 | 0.65 | (0.36,1.17) | 0.15 | 0.98 | (0.67,1.43) | 0.9 | 0.96 |
| Selenium | T1/T2 | 1.51 | (0.84,2.74) | 0.17 | 1.16 | (0.52,2.56) | 0.72 | 1.38 | (0.86,2.21) | 0.19 | 0.62 |
|  | T3 | 1.31 | (0.61,2.83) | 0.48 | 0.74 | (0.36,1.52) | 0.42 | 0.97 | (0.58,1.64) | 0.91 | 0.96 |
| Thallium | T1/T2 | 0.56 | (0.31,1.01) | 0.054 | 0.92 | (0.48,1.77) | 0.81 | 0.7 | (0.45,1.09) | 0.11 | 0.53 |
|  | T3 | 0.81 | (0.5,1.3) | 0.38 | 1.05 | (0.65,1.71) | 0.83 | 0.92 | (0.66,1.29) | 0.63 | 0.96 |
| Tin | T1/T2 | 1.15 | (0.85,1.56) | 0.38 | 0.76 | (0.45,1.27) | 0.29 | 1.03 | (0.79,1.34) | 0.83 | 0.86 |
|  | T3 | 1.07 | (0.82,1.39) | 0.62 | 0.76 | (0.54,1.08) | 0.13 | 0.95 | (0.77,1.17) | 0.61 | 0.96 |
| Zinc | T1/T2 | 0.95 | (0.65,1.4) | 0.81 | 0.93 | (0.55,1.58) | 0.78 | 0.94 | (0.69,1.29) | 0.72 | 0.82 |
|  | T3* | 0.76 | (0.54,1.06) | 0.11 | 0.86 | (0.59,1.26) | 0.45 | 0.8 | (0.62,1.03) | 0.09 | 0.82 |

*Symbols: ** meta-analysis p-value < 0.05. * meta-analysis p-value < 0.1.*

**Figure S1.** Gestational age at urine sample collection, separated by the trimester 1&2 pregnancy and trimester 3 pregnancy timepoints in A) EARLI cohort and B) MARBLES cohort.

| 1. **EARLI**   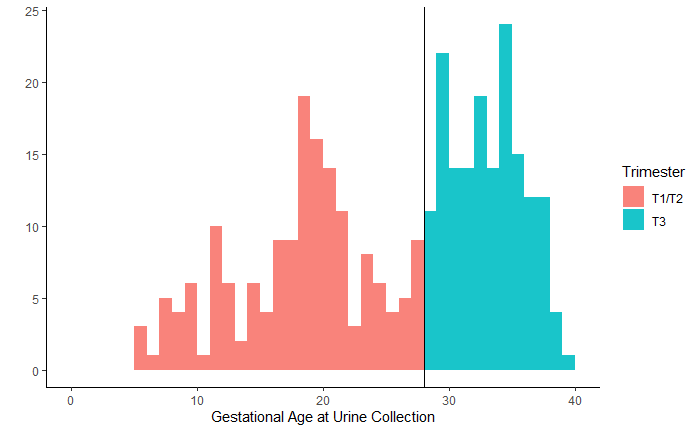 |
| --- |
| 1. **MARBLES**   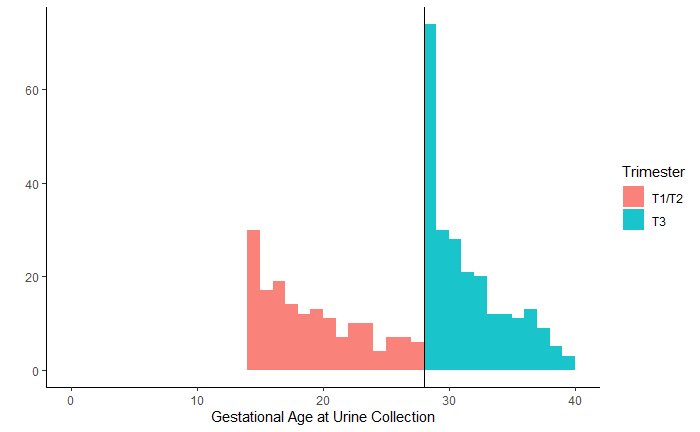 |

**Figure S2.** Inclusion and exclusion criteria flowchart for participants (N) and samples.


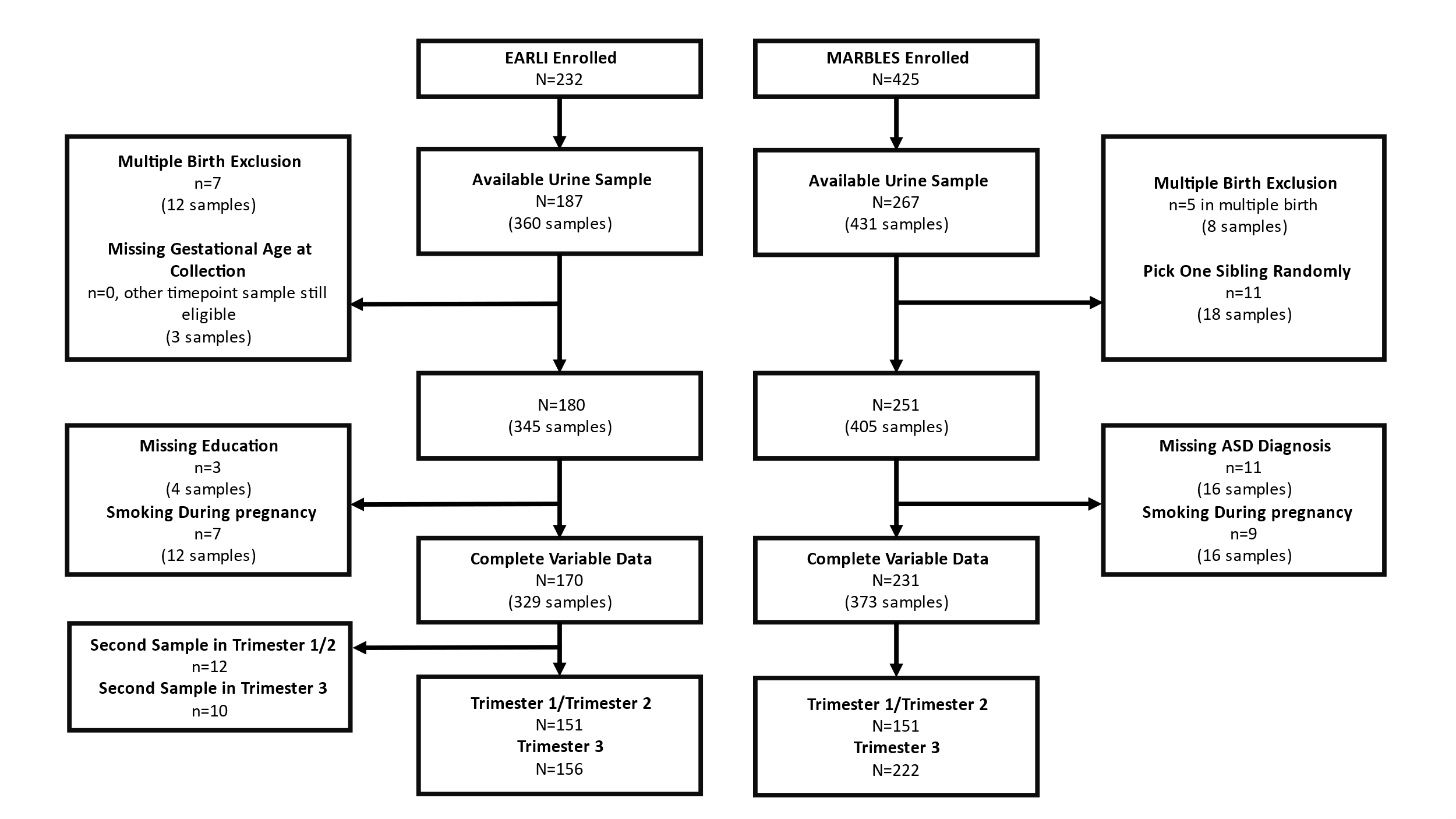


**Figure S3.** Spearman correlations of urinary metals concentrations, measured during T3 pregnancy, stratified by cohort. The upper right triangle shows the EARLI cohort. The lower left triangle shows the MARBLES cohort. Metals are represented by their chemical symbol along the diagonal.


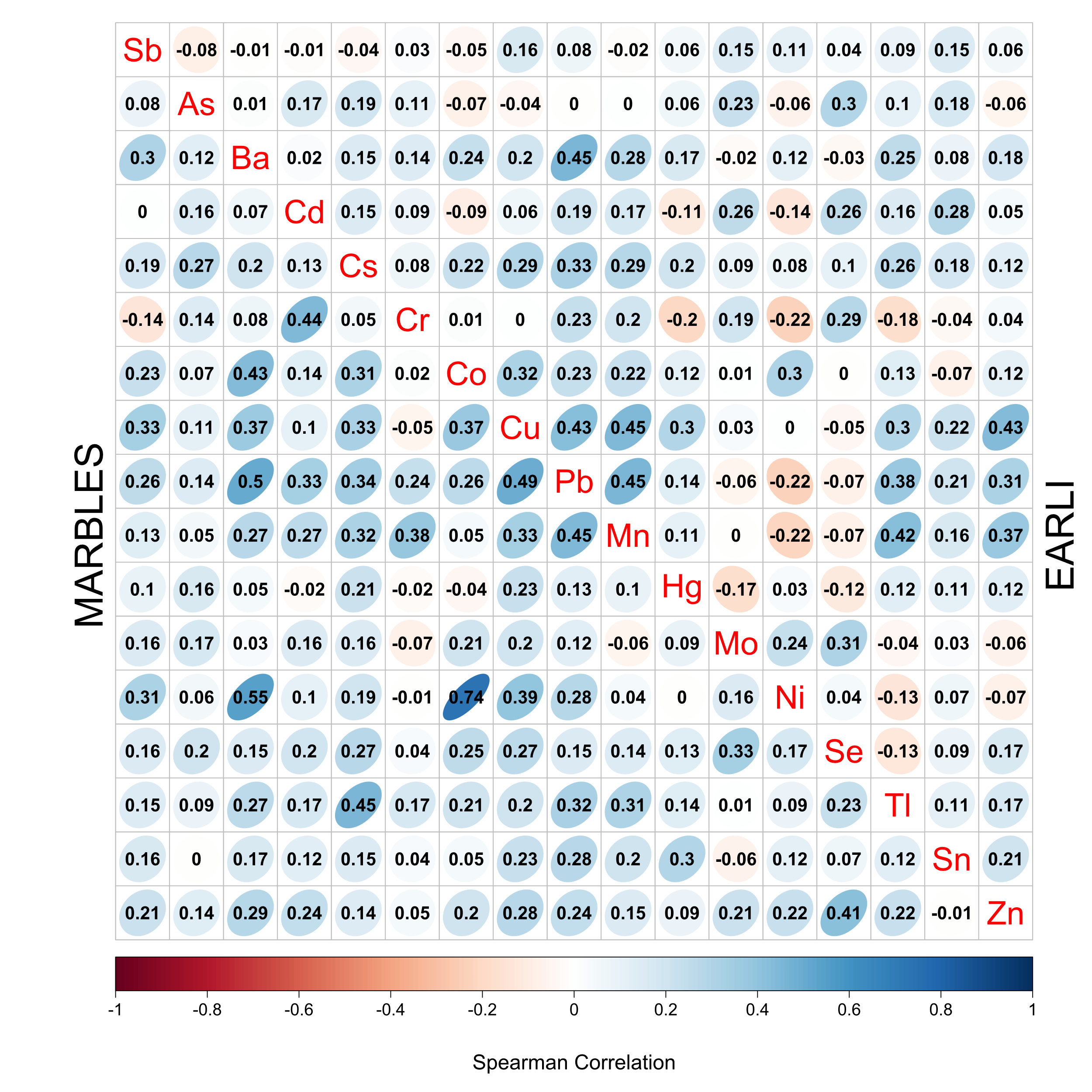


*Acronyms: Early Autism Risk Longitudinal Investigation (EARLI), Markers of Autism Risk in Babies-Learning Early Signs (MARBLES)*

**Figure S4**. Adjusted associations between pregnant participant blood metals concentrations and infant neurodevelopmental status in the Early Autism Risk Longitudinal Investigation (EARLI). **A)** Comparison between autism spectrum disorder relative to typically developing. **B)** Comparison between non-typically developing relative to typically developing. Relative risk ratios are reported for a doubling in concentration.

| **A.**  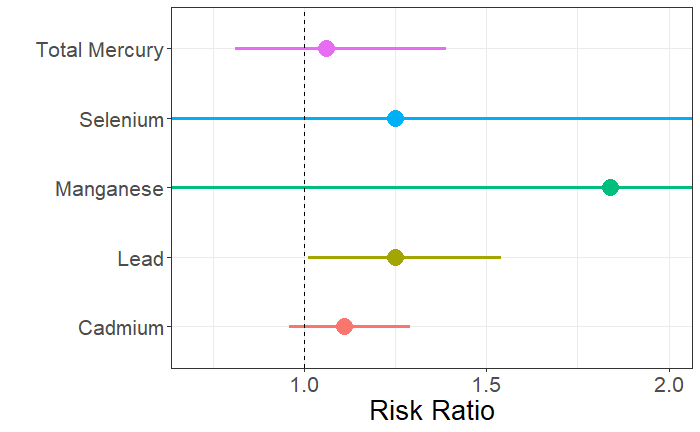 | **B.**  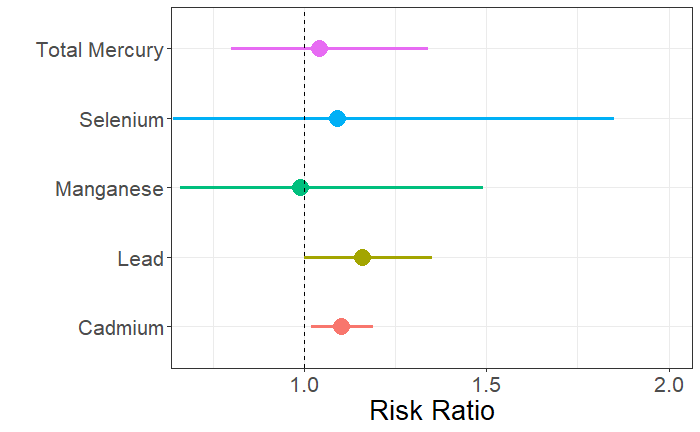 |
| --- | --- |
